# More π, please: What drives the formation of unsaturated molecules in the interstellar medium?†

**DOI:** 10.1039/d4sc07986h

**Published:** 2025-01-02

**Authors:** Jhoan Londoño-Restrepo, Santiago Gómez, Heidy M. Quitián-Lara, Felipe Fantuzzi, Albeiro Restrepo

**Affiliations:** a Instituto de Física, Universidad de Antioquia Calle 70 No. 52-21 Medellín Colombia; b Instituto de Química, Universidad de Antioquia Calle 70 No. 52-21 Medellín 050010 Colombia albeiro.restrepo@udea.edu.co; c Department of Physics, Chemistry and Biology (IFM), Linköping University Linköping SE-581 83 Sweden; d Max Planck Institute for Extraterrestrial Physics Gießenbachstraße 1 85748 Garching Germany heidyql@mpe.mpg.de; e Physics and Astronomy, School of Engineering, Mathematics and Physics, University of Kent Park Wood Rd Canterbury CT2 7NH UK; f Chemistry and Forensic Science, School of Natural Sciences, University of Kent Park Wood Rd Canterbury CT2 7NH UK f.fantuzzi@kent.ac.uk

## Abstract

We present a computational investigation into the fragmentation pathways of ethanolamine (C_2_H_7_NO, EtA), propanol (C_3_H_8_O, PrO), butanenitrile (C_4_H_7_N, BuN), and glycolamide (C_2_H_5_NO_2_, GlA)—saturated organic molecules detected in the interstellar medium (ISM), particularly in the molecular cloud complex Sagittarius B2 (Sgr B2) and its molecular cloud G+0.693-0.027. Using electron-impact ionization data and Born–Oppenheimer molecular dynamics simulations, we investigate how cosmic rays, cosmic-ray-induced UV fields, and shock-induced heating can induce the fragmentation of these molecules, resulting in the formation of unsaturated species with extended π-bond networks. Despite the attenuation of external UV radiation in G+0.693-0.027, these energetic processes are capable of driving partial transformations of saturated into unsaturated molecules, supporting the coexistence of species like EtA and GlA alongside unsaturated nitriles such as cyanoacetylene (HC_3_N), cyanopropyne (CH_3_C_3_N), and cyanoallene (CH_2_CCHCN). Our findings underscore the significance of high-energy mechanisms in enhancing chemical complexity within molecular clouds and offer insights into the pathways that govern the evolution of organic molecules in the ISM.

## Abstract 2

We computationally investigated the fragmentation pathways of saturated molecules detected in the interstellar medium, demonstrating how high-energy processes drive their transformation into unsaturated species with extended π-bond networks.

## Graphical abstract

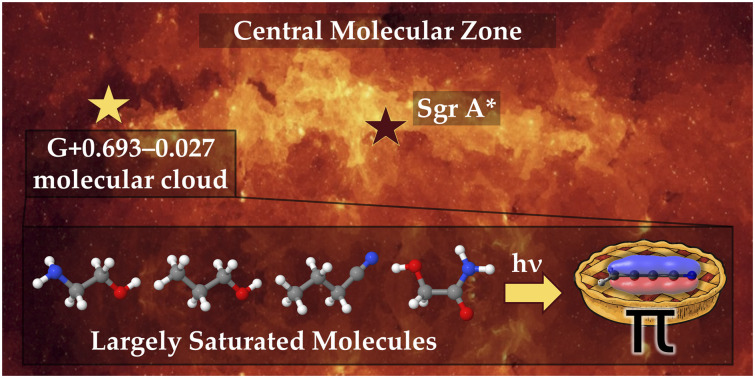

## Introduction

The observation of neutral and charged molecular species in the interstellar medium (ISM) has become almost routine in recent times. Indeed, to date, more than 320 different molecules spanning a wide range of complexity have been reported (see *e.g.* the Cologne Database for Molecular Spectroscopy CDMS website).^1^ Interestingly, a significant portion of the molecular species observed in the ISM is highly unsaturated, containing multiple π bonds or extended conjugated systems.^2,3^ At first glance, this might seem contradictory given the high abundance of hydrogen in the universe, which might be expected to favor the formation of saturated molecules through hydrogenation reactions. However, in molecular clouds, hydrogen primarily exists in its molecular form (H_2_), which is relatively unreactive under typical interstellar conditions.^4,5^ The formation and persistence of unsaturated molecules, therefore, depend heavily on local physical and chemical conditions, such as temperature, density, and radiation fields.^6^ For example, cold, dense regions often support diverse chemistries: grain-surface reactions favor the formation of saturated species like methanol and ammonia,^7^ while gas-phase processes drive the synthesis of unsaturated carbon chains and radicals.^8,9^ Conversely, star-forming hot cores and hot corinos typically harbor terrestrial-like, hydrogen-rich molecules such as esters, ethers, and alcohols, reflecting the role of higher temperatures in facilitating the desorption of saturated species from grain surfaces.^10,11^

Several reaction mechanisms, supported by substantial experimental evidence, have been proposed to explain the formation of hydrogen-deficient molecules in the harsh environments where they are found.^3,8,9,12–14^ These conditions, considered extreme by Earth laboratory standards, typically involve low temperatures and pressures combined with exposure to high-energy processes including cosmic rays and X-rays. Two main chemical processes, each with their own ramifications, appear to govern the production of local π bonds and their subsequent accumulation into highly unsaturated carbon chains: gas phase ion–molecule reactions and recombinative dissociation.^2,3,15^ Moreover, cosmic rays and X-rays can penetrate deep into dense molecular clouds, initiating ionization and fragmentation processes that contribute to the formation of unsaturated molecules.^16^

In this context, using molecular dynamics simulations and transition state theory, Paranjothy and coworkers^14^ proposed possible mechanisms for the formation of these large unsaturated molecules *via* neutral–ion reactions, specifically between the C_2_^−^ anion and acetylene (C_2_H_2_) as precursors. Dust particle and proton transfer reactions, as well as top-down mechanisms, have also been studied. Most recently, Tajuelo-Castilla *et al.*^17^ argued that UV-induced chemistry in the ISM plays a significant role, particularly in the dehydrogenation of carbonaceous cosmic dust.^18–20^ However, in dense molecular clouds like those found in the central molecular zone (CMZ) of the Milky Way and those close to the solar neighbourhood, external UV radiation is significantly attenuated, and other high-energy processes such as cosmic rays, cosmic-ray-induced UV fields,^21,22^ and X-rays become more influential in driving chemical reactions.

The CMZ, encompassing the central 600 pc of the Milky Way, contains approximately 5–10% of the total molecular gas reservoir in the Galaxy, amounting to 3–5 × 10^7^ M_⊙_ concentrated in this region.^23^ Due to its extreme conditions, the CMZ has a molecular gas surface density almost two orders of magnitude higher than typical levels found in the Galactic disk,^23,24^ providing a unique environment for studying molecule formation under extreme conditions. The CMZ is characterized by warm-hot, highly magnetized, dense, and turbulent molecular gas.^25–28^ This gas is constantly exposed to radiation from cosmic rays, X-rays, and UV photons originating from the Galactic Center, massive stars, and young stellar clusters, which can ionize and modify the region over tens of parsecs.^29,30^ A particularly notable feature of the CMZ is the asymmetric distribution of molecular gas.^25,31^ These asymmetries modify the effective extinction values compared to observed values,^32^ making it challenging to precisely determine the radiative flux impacting the different molecular clouds in this region.

One of the key regions of astrochemical interest within the CMZ is Sgr B2 (Sagittarius B2), a large, dense, and cold molecular cloud that spans approximately 36 pc and is located about 100 pc from Sgr A*, the supermassive black hole at the Galactic Center.^33,34^ Sgr B2 is situated in this highly dynamic environment, characterized by intense turbulence, elevated cosmic-ray fluxes, and strong X-ray fields, all of which play significant roles in shaping star-formation processes and chemical composition.^35–37^ The massive size of Sgr B2 means that it is not an isotropic medium, and local conditions can change drastically within distinct subdomains. For example, the average atomic density of Sgr B2 is about 3000 cm^−3^, but it can reach as high as 10^5^ cm^−3^ for H_2_ alone, with temperatures up to 300 K.^38^ Additionally, Sgr B2 is characterized by enhanced cosmic-ray ionization rates—estimated to be 10^−15^–10^−14^ s^−1^, 100–1000 times higher than in the Galactic disk^22,39,40^—and the presence of low-velocity shocks resulting from cloud–cloud collisions.^36,41,42^ These cosmic rays and shocks can dominate the heating and ionization processes within the molecular clouds, even over traditional mechanisms like photoelectric heating from UV radiation.^35,37^

A notable subdomain of Sgr B2 is G+0.693-0.027 (G+0.693 for short), which is ≈2.4 pc in diameter and where more than 120 molecular species have been detected,^43^ including prebiotic compounds. G+0.693 is considered a quiescent giant molecular cloud, showing no clear signs of active star formation, yet it exhibits a rich and complex chemistry.^35,41^ This complexity is thought to be driven by non-thermal desorption processes induced by low-velocity shocks and elevated cosmic-ray fluxes, which release molecules from dust grain mantles into the gas phase.^35,36,44^

**Fig. 1:**
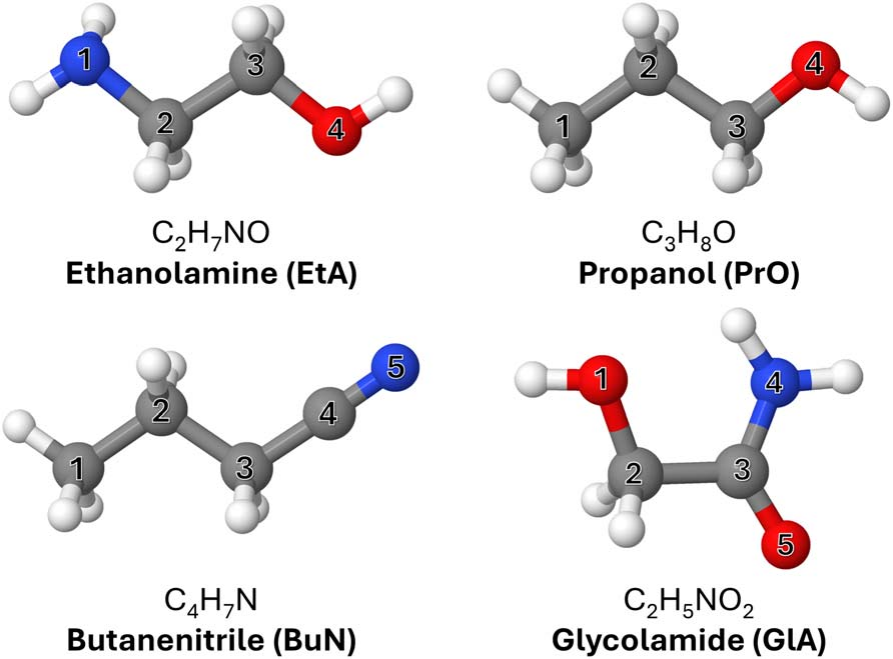
Molecules investigated in this work and the corresponding atomic labels. Heavy atoms are labeled differently in each case.

Four of the complex organic molecules detected either in G+0.693 or Sgr B2—namely ethanolamine (C_2_H_7_NO, EtA), glycolamide (C_2_H_5_NO_2_, GlA), propanol (C_3_H_8_O, PrO), and butanenitrile (C_4_H_7_N, BuN)—are particularly relevant to the present work (Fig. 1). C=O and C≡N bonds aside, these molecules are highly saturated having single C–C, C–N, C–O bonds as well as terminal C–H, N–H and O–H bonds. In 2009, Belloche and coworkers^45^ detected the *anti* conformer of butanenitrile (also known as *n*-propyl cyanide) in Sgr B2 using microwave data, and argued that there is a high degree of certainty that the *gauche* conformer is also present. In 2021, Rivilla and coworkers^46^ detected neutral ethanolamine in G+0.693. This molecule, a key phospholipid component, has been systematically investigated through irradiation and thermal processing of ice analogs under interstellar-like conditions.^47,48^ In 2022, Rivilla and co-authors identified two conformers of propanol in G+0.693,^49^ followed by the detection of glycolamide—a glycine isomer—in the same source in 2023.^22^ The presence of these saturated molecules in an environment characterized by elevated cosmic-ray fluxes and shocks indicates that their formation or release into the gas phase may occur *via* non-thermal desorption processes driven by these energetic phenomena.^35,36^ Notably, several unsaturated molecules, including cyanoacetylene (HC_3_N), cyanopropyne (CH_3_C_3_N), and cyanoallene (CH_2_CCHCN), have also been detected in G+0.693-0.027, highlighting the region's chemical diversity.^50^ Understanding how saturated molecules can persist, or how they may fragment under the influence of cosmic rays, cosmic-ray-induced UV fields,^21,22^ and X-rays, is crucial for explaining the observed abundances of unsaturated species in such regions.

In this work, we investigate suitable conditions and mechanisms that facilitate the formation of highly unsaturated species to explain their puzzling abundances. To that end, we perform a series of Born–Oppenheimer molecular dynamics (BOMD) simulations to model the fragmentation pathways of four complex organic molecules detected in Sgr B2 (Fig. 1), namely ethanolamine, glycolamide, propanol, and butanenitrile, under high-energy conditions akin to those in G+0.693. The results are compared with experimental data available in the literature. Understanding these processes not only sheds light on the intricate chemistry of the interstellar medium but also offers insight into the potential pathways for the formation of prebiotic molecules in space.

## Methods

We retrieved the experimental mass spectra of EtA, BuN and PrO from the NIST database.^51^ In turn, the spectrum of GlA was obtained from the John Wiley & Sons, Inc. SpectraBase.^52^ Simulations of fragmentation pathways for the four parent molecules were carried out using the QCxMS package.^53,54^ This software performs conformational sampling of the neutral molecules *via* BOMD, yielding snapshots that are then used as initial structures for a desired number of fragmentation dynamics. To test the stability of highly unsaturated fragments, we also ran BOMD calculations using these fragments as initial structures in simulations under the same conditions as those of the parent molecules. We used the extended semiempirical tight-binding model GFN2-xTB^55,56^ to compute the electronic structure during molecular fragmentation. Our choice is supported by its known accuracy, yielding reliable results when tested on an extensive set of organic and inorganic molecules.^57,58^

A total of 12 000 dissociation dynamics were computed from the selected snapshots for periods of 50 and 100 ps (6000 dynamics each), with an integration time step of 0.25 fs. The impact ionization energy was set to 288 eV, which corresponds to the resonance energy of the 1s electrons of the C atom. The ions obtained during the BOMD runs were further optimized to potential energy surface minima, as characterized by their computed analytical Hessians. This was done with the aid of second-order Møller–Plesset perturbation theory (MP2) and the 6-311++G(d, p) basis set. For cases where isomeric forms of the same *m*/*q* ratio were obtained, single-point coupled-cluster calculations at the CCSD(T) level with the aforementioned basis set were used to determine their relative stabilities. For the ions that followed the main fragmentation routes *via* C–C bond breaking, we analyzed the structures and chemical bonding situations as a function of the length of that bond until dissociation.

We calculated total energies, Wiberg bond indexes (WBI),^59^ and their first derivatives with respect to the nuclear position. For the well defined minima, we computed the electron localization function (ELF)^60,61^ and provided their heat maps at the appropriate planes, as well as the number of electrons in the basins corresponding to the C–C bonds. These descriptors were supplemented with adaptive natural density partition (AdNDP)^62^ orbitals and populations, along with plots of the spin densities. Gaussian 09,^63^ Multiwfn 3.8,^64^ and NBO 6.0 ^65^ were used for the electronic structure calculations and postprocessing analyses. JMol,^66^ VESTA,^67^ and Avogadro^68^ were used to draw molecular structures, ELF plots, AdNDP orbitals, spin densities, and molecular orbitals. Finally, an inspection of the ground and excited state potential energy curves of ionized GlA following the C2–C3 dissociation pathway was also conducted, with the calculations performed using Orca 5.0.4.^69^

## Results and discussion

### Comparison with experimental spectra

We begin by comparing the experimental spectra with those obtained from our simulations, and contextualizing these results for photoionization triggered by interstellar radiation. Our goal is not to faithfully reproduce the experimental data, but to verify if the fragments are appropriately captured in our simulations. This contributes to our primary objective: providing a mechanistic understanding of the pathways leading to molecular fragmentation and the nature of the fragments in an astronomical context. All experimental spectra from the literature were obtained using 70 eV electron ionization (EI). At this point, it is worth comparing the fragmentation profiles obtained by EI with those from photoionization methods.

EI typically results in a higher number of fragment ions and a more complex mass spectrum compared to early photoionization experiments using atomic emission lamps with energies typically below 20 eV, where the ionization process is generally softer and produces fewer fragments.^70^ However, several studies have reported similarities between the shapes of EI spectra at 70 eV and photoionization spectra using a He I lamp (21.21 eV).^71,72^ Furthermore, at energies in the soft X-ray range, photo-induced fragmentation can be even more pronounced than at 70 eV EI.^72–76^ In the astrochemical context, X-rays have greater penetration power than UV photons, enabling them to influence the chemistry deeper inside molecular clouds. This justifies our choice of using impact energies of 288 eV in our simulations.

**Fig. 2:**
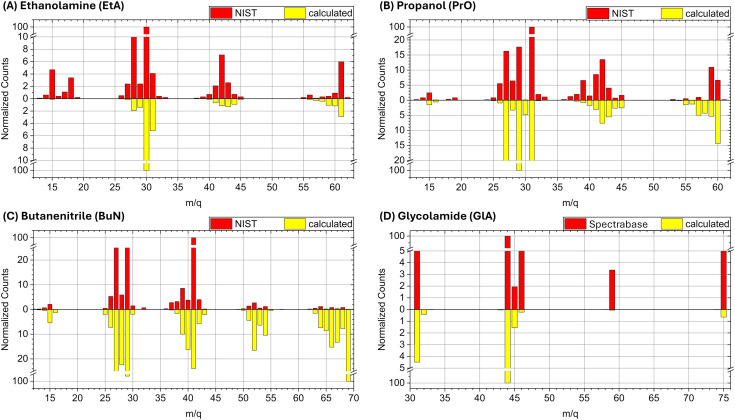
Experimental and calculated spectra for (A) ethanolamine (EtA), (B) propanol (PrO), (C) butanenitrile (BuN), and (D) glycolamide (GlA).

Next, we discuss the comparison of the spectra on a case-by-case basis, starting with ethanolamine (Fig. 2A). The experimental peaks below *m*/*q* = 27 are not obtained in the computations because the running times of the BOMD simulations are just too short to allow the cascade of fragmentation events leading to the smaller fragments. The cluster of peaks in the *m*/*q* = 14 to 18 range are likely due to ionic forms of CH_2_, CH_3_, NH_2_, OH, and H_2_O. Notice that the three main fragmentation routes discussed below, which involve breaking of the N1–C2, C2–C3, and C3–O4 bonds and further loss of hydrogen, do not produce species lighter than *m*/*q* = 27. Thus, the BOMD simulations are quite adequate for this analysis.

For propanol (Fig. 2B) the computed mass spectrum offers perhaps the best match with the experimental data, both in terms of intensities and fragmentation patterns, among the four molecules studied here. Even the small peak at *m*/*q* = 15, corresponding to the CH_3_^+^ cation, which results from the rupture of the terminal C1–C2 σ bond, is captured by the BOMD simulations. The main fragmentation route, as discussed below, involves the rupture of the central C2–C3 σ bond.

For butanenitrile (Fig. 2C), our computational results show a heavy population in the vicinities of the parent ion, in contrast to the low yield of these peaks in the experimental spectrum. We attribute this discrepancy to insufficient simulation times to properly capture the cascade reactions. Nonetheless, all other features are accurately reproduced, including the *m*/*q* = 15 signal, which corresponds to the CH_3_^+^ cation resulting from the rupture of the terminal C1–C2 σ bond.

Finally, for glycolamide (Fig. 2D), the spectrum is the simplest and is accurately reproduced by the calculations in terms of peak positions. However, we do not reproduce the relative intensities of masses above *m*/*q* = 46, although these signals are notably small in the experimental spectrum compared to the dominant *m*/*q* = 44 peak. The largest mismatch is observed for the *m*/*q* = 59 signal, likely attributed to [C_2_H_5_NO]^+^ (resulting from a loss of oxygen from the parent ion). In the experimental spectrum, this ion has a normalized count of around 3.4, whereas it is captured by the BOMD simulations with very few counts. Additionally, it is important to note that the break in the vertical scale for this spectrum is different from the other cases, which might exaggerate the perceived differences in intensity when compared to the other spectra.

### Main fragmentation paths

**Fig. 3:**
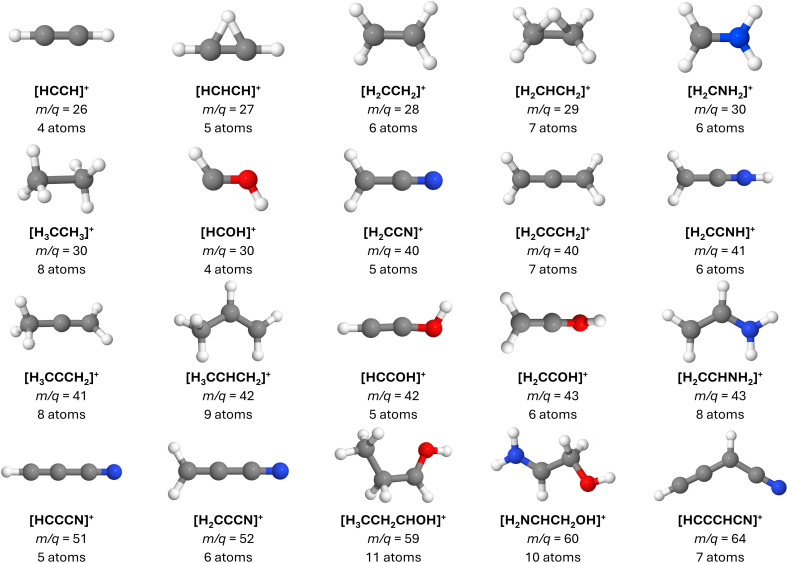
Main ionic fragments produced by the dissociative ionization of the molecules investigated herein and that were still not identified in the interstellar medium. Notably, in a few cases, the neutral counterparts of these ions have already been detected. For each case, the molecular formula, *m*/*q* ratio, and the total number of atoms are specified. Notice that most of the species featured herein possess at least one π bond. See text for details.

In this section, we briefly discuss the primary fragmentation paths observed in our BOMD calculations. For an in-depth analysis, please see the ESI file, including Fig. S1 to S5 and Tables S1, S2.† A comprehensive list of the main cationic fragments obtained in this study is presented in Table 1. Additionally, the key ionic fragments generated during the dynamics that have not yet been identified in the ISM are illustrated in Fig. 3.

**Table 1:** List of cationic fragments obtained through the BOMD simulations. Δ*E* represents the relative energy of distinct isomers at the CCSD(T)/6-311++G(d, p)//MP2/6-311++G(d, p) level of theory. Columns 5–8 indicate the detection of the fragment in the fragmentation dynamics of the respective starting compound, where Y denotes detection above the normalized intensity of 0.2. Numbers within parentheses indicate the number of counts including both the 50 ps and 100 ps dynamics. Abbreviations: EtA (ethanolamine), PrO (propanol), BuN (butanenitrile), GlA (glycolamide). In column 9, Y denotes that the cationic fragment was detected in the ISM, while Ne indicates the detection of its neutral form. In column 10, the same rationale applies, but the detections are specifically within Sgr B2

*m*/*q*	Molecular formula	Ionic species	Δ*E*	Presence in				Detected in ISM	Detected in Sgr B2
				EtA	PrO	BuN	GlA		
15	CH_3_	[CH_3_]^+^	—	—	Y (59)	Y (134)	—	—	—
26	C_2_H_2_	[HCCH]^+^	—	—	Y (38)	Y (176)	—	Ne^77^	—
27	C_2_H_3_	[HCHCH]^+^	—	Y (9)	Y (38)	Y (1870)	—	—	—
27	CHN	[HCN]^+^	—	Y (6)	—	—	—	Ne^78^	—
28	C_2_H_4_	[H_2_CCH_2_]^+^	—	—	Y (129)	Y (552)	—	Ne^79^	—
28	CH_2_N	[HCNH]^+^	0.0	Y (203)	—	Y (69)	—	Y^80^	Y^80^
28	CH_2_N	[H_2_CN]^+^	70.5	Y (4)	—	—	—	Ne^81^	Ne^81^
29	C_2_H_5_	[H_2_CHCH_2_]^+^	—	—	Y (4476)	Y (2460)	—	—	—
29	CH_3_N	[HCNH_2_]^+^	0.0	Y (54)	—	—	—	—	—
29	CH_3_N	[H_2_CNH]^+^	3.4	Y(65)	—	—	—	—	—
29	CHO	[HCO]^+^	—	Y (22)	Y (129)	—	—	Y^82^	Ne^83^
30	C_2_H_6_	[H_3_CCH_3_]^+^	—	—	Y (108)	—	—	—	—
30	CH_2_O	[H_2_CO]^+^	0.0	Y (10)	Y (31)	—	—	Ne^84^	Ne^84^
30	CH_2_O	[HCOH]^+^	7.4	Y (9)	Y (68)	—	—	—	—
30	CH_4_N	[H_2_CNH_2_]^+^	—	Y (9982)	—	—	—	—	—
31	CH_3_O	[H_2_COH]^+^	—	Y (543)	Y (3298)	—	Y (551)	Y^85^	Y^85^
31	CH_5_N	[H_3_CNH_2_]^+^	—	Y (34)	—	—	—	Ne^86^	Ne^86^
32	CH_4_O	[H_3_COH]^+^	—	—	—	—	Y (21)	Ne^87^	Ne^87^
38	C_3_H_2_	[HCCCH]^+^	—	—	Y (11)	Y (42)	—	—	—
39	C_3_H_3_	[H_2_CCCH]^+^	—	—	Y (37)	Y (187)	—	Y^88^	—
39	C_2_NH	[HCCN]^+^	—	—	—	Y (38)	—	Ne^89^	—
40	C_2_H_2_N	[H_2_CCN]^+^	0.0	—	—	Y (217)	—	Ne^90^	Ne^90^
40	C_2_H_2_N	[HCCNH]^+^	24.4	—	—	Y (57)	—	—	—
40	C_3_H_4_	[H_2_CCCH_2_]^+^	0.0	—	—	Y (77)	—	—	—
40	C_3_H_4_	[H_3_CCCH]^+^	14.0	—	—	Y (56)	—	Ne^91^	Ne^91^
41	C_2_H_3_N	[H_2_CCNH]^+^	0.0	Y (29)	—	Y (415)	—	Ne^92^	Ne^92^
41	C_2_H_3_N	[HCCNH_2_]^+^	19.4	Y (17)	—	—	—	—	—
41	C_2_H_3_N	[H_2_CNCH]^+^	20.5	—	—	Y (22)	—	—	—
41	C_2_H_3_N	[H_3_CCN]^+^	60.9	—	—	Y (26)	—	Ne^93^	Ne^93^
41	C_2_HO	[HCCO]^+^	—	Y (5)	Y (25)	—	—	Ne^94^	—
41	C_3_H_5_	[H_3_CCCH_2_]^+^	—	—	Y (100)	Y (126)	—	—	—
42	C_2_H_2_O	[H_2_CCO]^+^	0.0	Y (19)	Y (66)	—	—	Ne^95^	Ne^95^
42	C_2_H_2_O	[HCCOH]^+^	45.1	Y (17)	Y (71)	—	—	—	—
42	C_2_H_4_N	[H_3_CCNH]^+^	0.0	Y (32)	—	—	—	—	—
42	C_2_H_4_N	[H_2_CCNH_2_]^+^	16.4	Y (40)	—	—	—	—	—
42	C_3_H_6_	[H_3_CCHCH_2_]^+^	—	—	Y (194)	Y (147)	—	—	—
43	C_2_H_3_O	[H_3_CCO]^+^	0.0	Y (18)	Y (29)	—	—	Y^96^	—
43	C_2_H_3_O	[H_2_CCOH]^+^	40.1	Y (30)	Y (176)	—	—	—	—
43	C_2_H_5_N	[H_2_CCHNH_2_]^+^	—	Y (69)	—	—	—	Ne^97^	Ne^97^
44	CH_2_NO	[H_2_NCO]^+^	—	—	—	—	Y (10 686)	Y^98,99^	Y^98^
45	CH_3_NO	[H_2_NCOH]^+^	—	—	—	—	Y (6)	—	—
51	C_3_HN	[HCCCN]^+^	—	—	—	Y (93)	—	Ne^100^	Ne^100^
52	C_3_H_2_N	[HCCCNH]^+^	0.0	—	—	Y (107)	—	Y^101^	—
52	C_3_H_2_N	[HCNCCH]^+^	17.7	—	—	Y (25)	—	Y^102^	—
52	C_3_H_2_N	[H_2_CCCN]^+^	36.7	—	—	Y (199)	—	Ne^103^	—
52	C_3_H_2_N	[c-HCCHCN]^+^	51.7	—	—	Y (35)	—	—	—
59	C_3_H_7_O	[H_3_CCH_2_CHOH]^+^	—	—	Y (246)	—	—	—	—
60	C_2_H_6_NO	[H_3_CCOHNH_2_]^+^	0.0	Y (1)	—	—	—	—	—
60	C_2_H_6_NO	[H_3_NCHCHOH]^+^	25.1	Y (3)	—	—	—	—	—
60	C_2_H_6_NO	[H_2_NCHCH_2_OH]^+^	27.7	Y (78)	—	—	—	—	—
60	C_2_H_6_NO	[H_3_NCH_2_CHO]^+^	29.9	Y (6)	—	—	—	—	—
60	C_2_H_6_NO	[H_2_CNH_2_CHOH]^+^	36.3	Y (4)	—	—	—	—	—
60	C_2_H_6_NO	[H_2_NCH_2_CHOH]^+^	48.2	Y (29)	—	—	—	—	—
64	C_4_H_2_N	[H_2_CCCCN]^+^	0.0	—	—	Y (52)	—	—	—
64	C_4_H_2_N	[HCCCHCN]^+^	1.9	—	—	Y (69)	—	—	—
64	C_4_H_2_N	[HCCCCNH]^+^	21.6	—	—	Y (30)	—	—	—

Ethanolamine (C_2_H_7_NO) fragmentation primarily involves breaking the C2–C3, C3–O4, and N1–C2 bonds. The most significant mass spectrum peak at *m*/*q* = 30 corresponds to [H_2_CNH_2_]^+^, indicating a preference for the positive charge on the nitrogen fragment. Peaks at *m*/*q* = 29, 28, 27 indicate sequential hydrogen loss, forming [H_2_CNH]^+^ and [HCNH_2_]^+^ (*m*/*q* = 29), [HCNH]^+^ (*m*/*q* = 28), and [HCN]^+^ (*m*/*q* = 27), with [HCO]^+^ (*m*/*q* = 29) also detected. Fragments in the *m*/*q* = 41–43 range are predominantly associated with N-bearing cations; however, the minor presence of O-bearing fragments within this range indicates the involvement of multiple fragmentation pathways. The *m*/*q* = 61 peak corresponds to cationic ethanolamine, and the *m*/*q* = 60 peak includes multiple [C_2_H_6_NO]^+^ isomers. An intense peak at *m*/*q* = 18, likely singly ionized water and protonated ammonia, was minimally detected during the BOMD span.

Propanol fragmentation, as identified by BOMD simulations, primarily involves breaking the C2–C3, C1–C2, and C3–O4 bonds. Additionally, a C–H bond rupture adjacent to the OH group leads to a *m*/*q* = 59 fragment. The C2–C3 bond breakage yields the intense [H_2_CHCH_2_]^+^ (*m*/*q* = 29) and [H_2_COH]^+^ (*m*/*q* = 31) peaks. These fragments further lose hydrogen atoms to form [HCCH]^+^ (*m*/*q* = 26) and [HCO]^+^ (*m*/*q* = 29). The C3–O4 bond breakage produces [H_2_CCHCH_3_]^+^, contributing to peaks around *m*/*q* = 40 and further degrading into [HCCCH_2_]^+^ (*m*/*q* = 39) and [HCCCH]^+^ (*m*/*q* = 38). The C1–C2 bond rupture leads to the *m*/*q* = 43 [H_2_CCOH]^+^ fragment, which can lose hydrogen atoms to form [HCCOH]^+^ and [H_2_CCO]^+^ (*m*/*q* = 42), contributing to the intense *m*/*q* = 42 peak with three structures.

Butanenitrile fragmentation, as revealed by BOMD simulations, involves three primary pathways. The main fragmentation routes include the breaking of the central C2–C3 bond, producing [H_2_CCN]^+^ (*m*/*q* = 40) and [H_2_CHCH_2_]^+^ (*m*/*q* = 29), and the terminal C1–C2 bond, yielding [CH_3_]^+^ (*m*/*q* = 15) and [H_2_CCH_2_CN]^+^ (*m*/*q* = 54). These fragments further degrade into highly unsaturated species like [HCCCNH]^+^, [H_2_CCCN]^+^, and [HCCCN]^+^. Another pathway involves structural isomerization followed by C3–C4 bond rupture, resulting in [H_3_CCCH_2_]^+^ (*m*/*q* = 41), which continues to lose hydrogen atoms to form [HCCCH]^+^. Additionally, rapid C2–C3 bond rupture produces [H_2_CCH_2_]^+^ (*m*/*q* = 28) and [H_2_CCNH]^+^ (*m*/*q* = 41), leading to further unsaturated fragments.

Glycolamide fragmentation, according to BOMD simulations, primarily involves the rupture of the C2–C3 bond. This process produces three main fragments. The most intense peak in both experimental and computed spectra is [H_2_NCO]^+^ (*m*/*q* = 44), followed by [H_2_COH]^+^ (*m*/*q* = 31), both detected in Sgr B2.^85,98^ Another fragmentation route produces cationic methanol, [CH_3_OH]^+^ (*m*/*q* = 32), also observed in Sgr B2 in its neutral form.^87^ Additionally, intramolecular proton transfer followed by C2–C3 bond rupture results in the [H_2_NCOH]^+^ fragment (*m*/*q* = 45), still elusive in the ISM.

Building on the analysis above, it is evident that the majority of the fragments generated through the fragmentation pathways are highly unsaturated, often featuring double or triple bonds and extended conjugated systems. We propose that the fragmentation of saturated molecules under high-energy processes, such as cosmic ray interactions, shock-induced heating, and secondary UV fields, plays an important role in the formation of unsaturated species in molecular clouds like G+0.693. Furthermore, this mechanism could potentially extend more broadly within the ISM, contributing to the observed prevalence of unsaturated molecules across diverse interstellar environments.

Lastly, while many of the observed fragments have already been detected, either in their ionic or neutral forms (see Table 1), we propose that the as-yet undetected fragments presented in Fig. 3, along with their neutral counterparts, are promising targets for future observational surveys. These fragments may play a role in interstellar chemistry and are compelling candidates for detection in a variety of astrophysical environments, especially when their parent molecules are present. Notably, once cationic species such as those in Table 1 are produced, subsequent pathways may result in the formation of neutral or alternative ionic molecules through diverse mechanisms. For example, [HCOH]^+^ can lose a proton or hydrogen atom to form HCO or [HCO]^+^, respectively, both of which are well-documented in various interstellar environments. Additionally, [HCOH]^+^ can undergo dissociative recombination upon electron capture,^104^ resulting in the dissociation of two neutral fragments.

Other cationic fragments in Table 1 similarly demonstrate the potential for such pathways. For instance, [HCNH]^+^ can yield neutral HCN, one of the most abundant and well-studied interstellar molecules.^78^ Similarly, [H_2_NCO]^+^, derived from the fragmentation of glycolamide, can form HNCO,^105^ which has been widely detected in interstellar environments, including Sgr B2.^106^ Another noteworthy example is [H_2_NCHCH_2_OH]^+^, the most frequently formed [C_2_H_6_NO]^+^ isomer following the ionization of ethanolamine according to our simulations, despite not being the thermodynamically most stable structure of this stoichiometry. Dissociative recombination of this species would lead to iminoethanol (C_2_H_5_NO),^107^ an isomer of acetamide that, unlike the latter,^108^ has yet to be discovered in the ISM.

These examples highlight the diversity of viable pathways by which cationic fragments can transition into stable neutral or ionic species, reinforcing the need to include hydrogen/proton-loss processes and molecular ion dissociative recombination in astrochemical models to improve our understanding of saturated and unsaturated chemical environments in the ISM.

### Electronic and structural properties of the main fragments

**Fig. 4:**
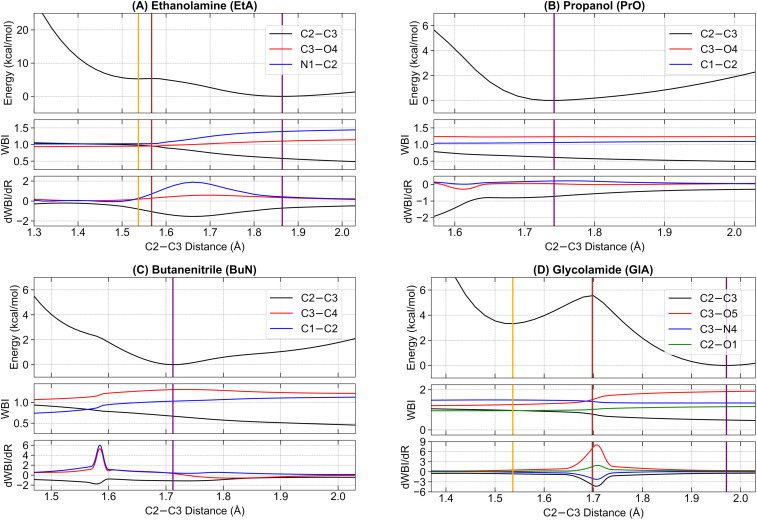
Electronic activity during the fragmentation of the C–C bonds in (A) ethanolamine (EtA), (B) propanol (PrO), (C) butanenitrile (BuN), and (D) glycolamide (GlA). The MP2/6–311++G(d, p) potential energy curves as a function of the C2–C3 distance are supplemented with the variation of the WBI values and their corresponding derivatives with respect to the nuclear coordinates. Solid vertical lines indicate the positions of the stationary points: orange for m1, brown for the transition state, and purple for either m2 or the single minimum (predissociation complexes) in PrO and BuN. Dashed vertical lines indicate the position of the transition states connecting m1 and m2 in EtA and GlA.

Next, we examine the initial stages of the dissociation process from the parent ions, starting at their radical cation equilibrium structures. We follow the potential energy curve generated from a relaxed scan, progressively increasing the central C2–C3 bonds of each system (see Fig. 1 for atom labeling details). This scan was chosen because the first step in the main fragmentation paths for the four molecules involves the rupture of the central C2–C3 bond. For each system, we track the electronic energy, the WBI of the C2–C3 and the two adjacent bonds involving heavy elements, and the first derivative of the WBI with respect to the nuclear coordinates, dWBI/d*R*. These plots are shown in Fig. 4.

The energy plots reveal a distinctive feature in EtA and GlA compared to the other two structures: the presence of two distinct energy minima along the dissociation path, each connected by a single transition state. We label the minima with the shortest C2–C3 bonds as EtA-m1 and GlA-m1, and those with elongated C2–C3 bonds as EtA-m2 and GlA-m2. The corresponding transition states are labeled EtA-TS and GlA-TS. The elongated C2–C3 bond minima EtA-m2 and GlA-m2 represent the lowest energy structures. The pure electronic energy barriers for the m1 → m2 conversion are relatively small: 0.06 kcal mol^−1^ for EtA and 2.26 kcal mol^−1^ for GlA. Using the Eyringpy program,^109^ we calculated rate constants on the order of 10^9^–10^11^ s^−1^ across the temperature range of 10–50 K (see Fig. S7 in the ESI†). These values correspond to half-lives in the range of *t*_1/2_ ≈ 1–100 ps, indicating that these reactions proceed extremely rapidly. This suggests that catalysts are unlikely to be required for the C–C dissociation to occur under these conditions.

To elucidate the nature of the observed barrier along the reaction coordinate of ionized GlA, additional theoretical analyses were performed using distinct single and multireference methods (see the ESI† for further details). These analyses consistently indicate that the barrier arises from structural reorganisations of the surrounding atoms rather than from an avoided crossing between electronic states. The results emphasise that ionization in this system involves vibrationally driven structural adjustments along the reaction coordinate, reinforcing the absence of state crossings as a key mechanistic feature.

For PrO and BuN, only a single minimum with an elongated C2–C3 bond was identified, though these bonds are shorter than those in the EtA-m2 and GlA-m2 systems. For clarity, whenever appropriate, EtA-m1 and GlA-m1 will be collectively referred to as m1, while EtA-m2 and GlA-m2 will be referred to as m2. Additionally, the lone minima in PrO and BuN, along with m2, will be collectively termed predissociation complexes.

**Fig. 5:**
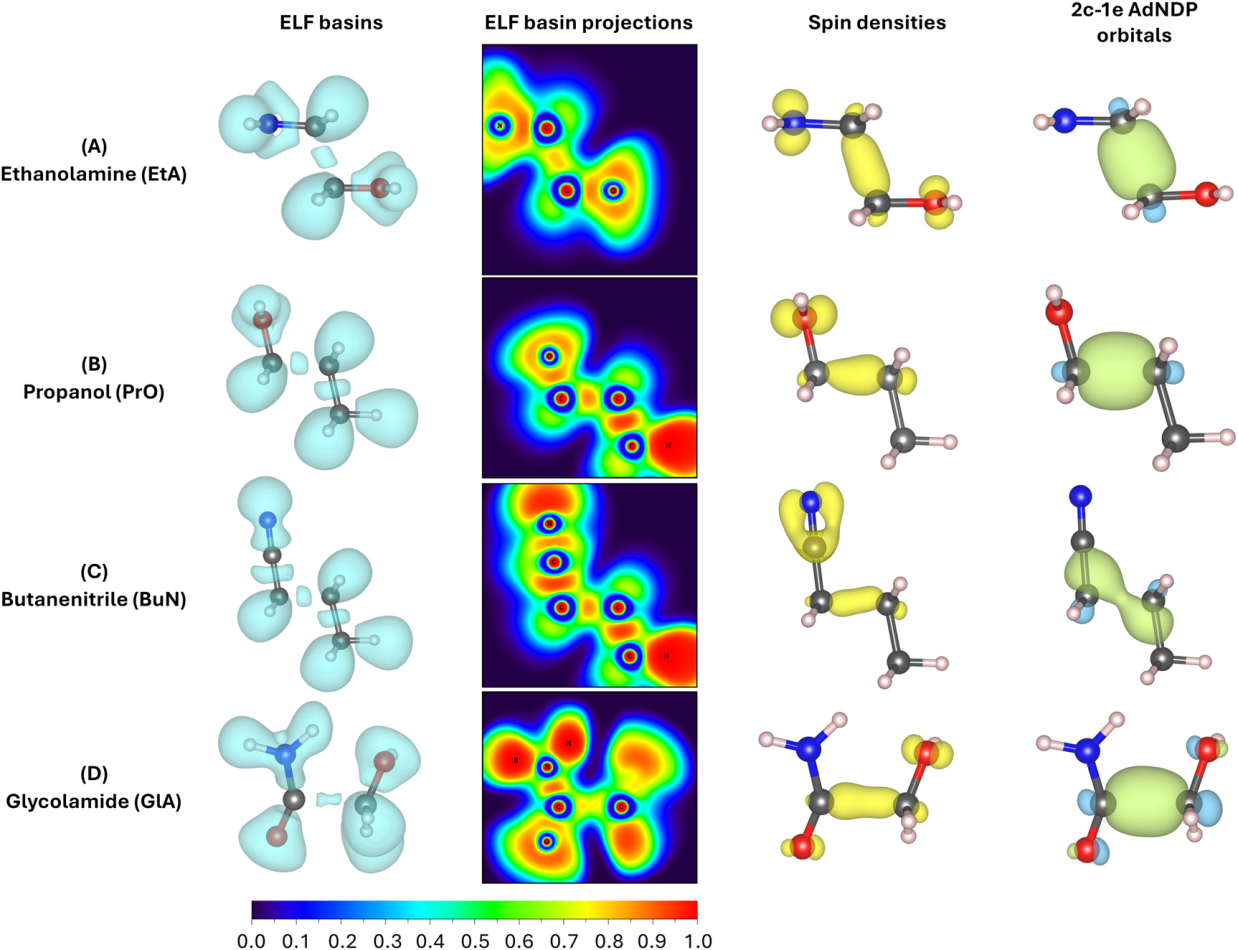
Evidence for the formation of two-center one-electron (2c–1e) bonds in the predissociation complexes of the radical cations in ethanolamine (A), propanol (B), butanenitrile (C), and glycolamide (D). The ELF basins are shown in the left column, the projections into the heavy atom planes are shown in the second column, the spin densities for the unpaired electrons are shown in the third column, and the 2c–1e AdNDP orbitals are shown in the right column.

In addition to Fig. 4, which shows the variation of the electronic energy, bond orders, and their derivatives as a function of the C2–C3 separation for all parent ions, Fig. 5 displays all mono and disynaptic basins, as well as the ELF heat maps in a carefully chosen heavy-atom plane. Table S3 in the ESI† lists the number of electrons and the corresponding WBI values for each basin and the AdNDP orbital associated with the C–C bonds in the predissociation complexes. We use this data to provide a formal picture of the bonding situation in the intermediates as follows.

First, notice that the C–C bonds in all predissociation complexes are remarkably similar. Indeed, all these C2–C3 bonds have WBIs in the vicinity of 0.5 and the number of electrons obtained from the integration of the ELF basins is very close to 1. According to the ELF, the C–C bond in GlA-m2 actually contains two monosynaptic basins, each with ≈0.5 electrons. These observations indicate a very unusual bonding pattern: just before fragmentation, local minima showing two-center one-electron (2c–1e) C–C bonds are involved in every case. Therefore, the radical cations prefer to delocalize the unpaired electron within the C–C bond rather than at specific atom locations, as is customarily depicted in chemical structures. We suggest the term “radical bonds” to describe these 2c–1e bonds, which not only have been the focus of recent theoretical investigations,^110–112^ but might also be widespread in organic radical cations.^113–115^ These radical bonds are beautifully seen in the 3D and 2D ELFs, in the α spin densities, and in the AdNDP orbitals shown in Fig. 5.

Heavy electronic activity—evidenced by strong peaks in the derivatives of the WBIs—early in the reaction path (Fig. 4) produces the single minimum with 2c–1e bonds in propanol and butanenitrile. Conversely, the formation of m1 involves little electronic activity, and the resulting C–C bond is well described as of a two-center two-electron (2c–2e) bond. As the reaction progresses, the electronic activity increases beyond the coordinate of m1, leading to m2. This involves overcoming a barrier and transforming the 2c–2e bonds into 2c–1e bonds in EtA and GlA.

Prior to the final dissociation into the fragments, the reduction of the C–C bond order is accompanied by subtle and/or moderate changes in other bonds, as shown in Fig. 4. The electronic activity for the m1 → TS → m2 process in both ethanolamine and glycolamide is schematized in Fig. S6 in the ESI.† This process involves a net reduction in the total electron density of the C–C bond *via* a gradual increase in the excess α spin density. This occurs through the transfer of one electron to the 2p orbital at the N center in ethanolamine and to the 2p orbital at the O center in glycolamide, resulting in the formation of C=N and C=O bonds in the fragmentation products.

This picture provides compelling evidence that, in m1, the radical is localized in the 2p orbitals of N and O, whereas in m2, the radical resides within the C–C bond, manifesting as a 2c–1e bond. In summary, the formation of a 2c–1e C–C bond appears to be a prerequisite for predissociation, irrespective of whether m1 is formed.

## Astrochemical implications

The ISM is home to a diverse range of molecular species, spanning both saturated and unsaturated systems. These two classes of molecules are typically associated with different formation mechanisms and distinct local environments. Saturated molecules are thought to form preferentially in warmer, denser regions such as hot cores and hot corinos near star-forming areas.^10,11^ Unsaturated molecules, on the other hand, are linked to colder regions where largely unreactive H_2_ dominates, enabling the accumulation of π-bonded structures through ion–molecule reactions and dissociative processes.^8,9^

The molecular cloud G+0.693, along with the larger Sgr B2 complex—one of the most chemically diverse regions in the interstellar medium—provides a remarkable environment for exploring the interplay between saturated and unsaturated chemistry, with the four molecules investigated in this study belonging to this rich chemical landscape.^116^ These systems, with their relatively simple structures and abundant hydrogen content, serve as clear indicators of the local chemical processes dominated by hydrogen-rich conditions. However, an intriguing aspect of G+0.693 is the simultaneous detection of unsaturated species, such as HC_3_N, CH_3_C_3_N, and CH_2_CCHCN.^50^

In G+0.693, external UV radiation is significantly attenuated due to the high column densities of molecular gas, which limits its role as a primary driver of photochemistry. This attenuation is characteristic of the CMZ, where the distribution of molecular gas is both asymmetric and highly dense,^25,31^ leading to variations in effective extinction across the region. However, despite the restricted role of external UV, G+0.693 is constantly exposed to other high-energy processes, such as cosmic rays, cosmic-ray-induced UV fields,^21,22^ X-rays, and shock-induced heating.^116^ These energetic processes may play a crucial role in shaping the chemistry of G+0.693.^35^ Cosmic-ray ionization rates in the CMZ are estimated to be significantly higher compared to those in the Galactic disk,^116^ facilitating the ionization and excitation of molecular species. Shock-induced heating, likely stemming from low-velocity shocks within the region, can liberate molecular precursors from dust grain mantles into the gas phase, enabling chemical reactions under otherwise unfavorable conditions. The detection of strong Fe Kα line emission in G+0.693 further supports the presence of ionizing agents, such as X-rays, which may act as an additional driver of chemical complexity.^117^ These combined high-energy processes provide alternative pathways for molecular transformation and fragmentation, potentially supporting the coexistence of both saturated and unsaturated molecules in this chemically rich molecular cloud.

Our findings suggest that these high-energy processes may indeed play a critical role in the chemical evolution of G+0.693, particularly in the formation of unsaturated species. Our computational simulations of the fragmentation pathways of saturated molecules under high-energy conditions reveal that the dominant products are unsaturated fragments, often featuring one or more π bonds, including cumulenic structures. For example, the fragmentation of glycolamide and butanenitrile consistently leads to products with extended π systems, demonstrating that high-energy processes such as cosmic ray ionization can induce bond-breaking and rearrangement events that favor the formation of unsaturated species.

We further propose that this phenomenon may extend to other giant molecular clouds, such as the Perseus Molecular Cloud and the Taurus Molecular Cloud (TMC), where the highest abundances of unsaturated species in the ISM have been observed. While the detection of X-ray sources in these regions is influenced by observational biases, the hypothesis is bolstered by the recent discovery of a 156 pc envelope around these clouds.^118,119^ This envelope encompasses diffuse X-rays with energies between 3–4 keV, likely resulting from pre-stellar feedback events or supernovae, which could have significantly modified the local gas chemistry over the past million years.^119^ These findings point to a broader applicability of the processes investigated in this study across different interstellar environments.

**Fig. 6:**
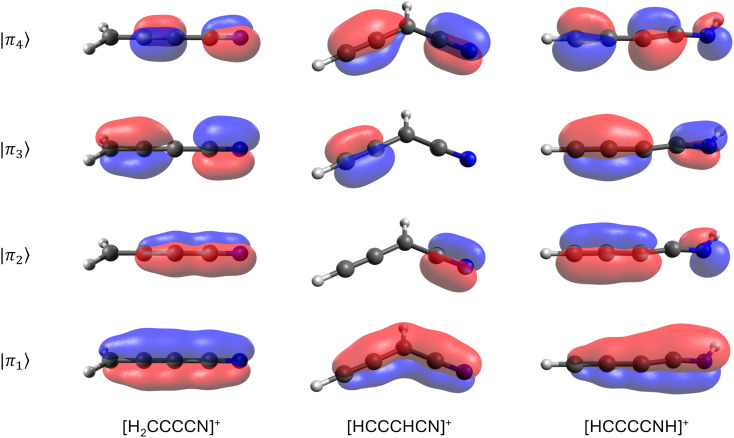
Extended π molecular orbitals for the *m*/*q* = 64 fragments in butanenitrile.

From the inventory of fragments listed in Table 1 obtained from the BOMD calculations, we derive the following numbers: a total of fifty-six main fragments are listed, of which twenty come from multiple sources. Among these unique fragments, twenty-six have one isolated π bond and twenty-two have multiple adjacent π bonds, with the largest number of adjacent π bonds being four (see the *m*/*q* = 64 fragments in Fig. 6). For more details, see Tables S1 and S2 in the ESI.† Interestingly, twelve species with one π bond and nine with multiple π bonds have already been detected as either singly charged species or neutral fragments in the ISM, including HCCCNH^+^, the protonated version of HCCCN. While HCCCNH^+^ was unambiguously detected in TMC-1,^101^ HCCCN was detected in Sgr B2.^91^ This highlights the potential of interstellar saturated molecules as precursors of unsaturated species through dissociation induced by high-energy events.

To test the stability of the unsaturated carbon chains, we ran 600 BOMD trajectories on each one of the three *m*/*q* = 64 fragments listed in Table 1. These ions consist of four adjacent π bonds (Fig. 6). The BOMD calculations were carried out under the same conditions as those for the parent molecular ions. The first notable result, clearly demonstrating the stability of the large carbon chains, is that, aside from the occasional loss of hydrogen atoms, the *m*/*q* = 64 fragments experience very little fragmentation, as shown in the calculated spectra (see Fig. S11 in the ESI†). Thus, once produced in the interstellar medium, highly unsaturated species have multiple pathways: they can either remain as stable units or if their abundances are sufficient, combine with other fragments to further grow the carbon chain.

Particularly striking is the observation that longer unsaturated chains are more abundant. For instance, Suzuki and coworkers^120^ found ratios of HC_5_N/HC_3_N of 3 ± 2 in TMC-1, while Dickens and coworkers^121^ reported HC_7_N/CCS ratios ranging from 3 to 5 in the same molecular cloud. These findings suggest that once formed, longer chains might be preferentially stabilized or replenished under interstellar conditions, allowing their accumulation in detectable quantities.

The reasons for the high stability provided to conjugated molecules have been a subject of active debate since the early days of quantum mechanics,^122–128^ and they remain a topic of discussion today.^129–137^ The roles of electronic kinetic energy and orbital contraction in covalent bonding have been recognized since the earliest treatments of the chemical bond.^112,138–142^ From a molecular orbital perspective, applying the simple model of a particle in a box to chemical bonds shows that the energy of each state is proportional to *n*^2^/*L*^2^. Therefore, the more delocalized the electron, the lower its destabilizing kinetic energy. As a result, extended networks of conjugated and adjacent π bonds are particularly low in energy. This formal argument aligns with the famous colloquial reasoning of Albert Szent-Györgyi when referring to oxidative metabolism, that “life is nothing but an electron looking for a place to rest”.

Our findings reveal that all fragmentation pathways studied consistently lead to the formation of fragments with the highest possible number of π bonds, including highly unsaturated species stabilized by extensive electron delocalization. These fragments, characterized by minimal hydrogen content and enhanced stability through conjugation, serve as essential building blocks in interstellar chemistry, emphasizing the importance of high-energy processes in the chemical evolution of molecular clouds.

## Conclusions

In summary, Born–Oppenheimer molecular dynamics calculations are used in this work to understand the fragmentation patterns of ethanolamine, propanol, butanenitrile, and glycolamide. These organic molecules are precursors to larger and more complex biomolecules and have been detected in Sgr B2, the vast molecular cloud near the supermassive black hole at the center of the Milky Way. Experimental mass spectra measured in Earth laboratories are consistently reproduced, both in the *m*/*q* ratios and in the intensities of the most important signals, thus giving strong support to our calculations. Several of the fragments produced in our calculations have also been detected in the interstellar medium, while the remaining fragments are suggested as possible targets for future detection. The main fragmentation routes for the four molecules studied involve the cleavage of C–C bonds. The rupture of the C–C bond in ethanolamine and glycolamide leads to two well-defined minima in the potential energy surface for the C–C separation. In contrast, for propanol and butanenitrile, a single minimum is observed. The electronic structures of the predissociation complexes indicate that the unpaired electron in the radical is not confined to a specific atom. Instead, it is transferred to the breaking C–C bond with small contributions from other molecular regions, leading to a 2c–1e bond situation. All radicals obtained in the cascade of fragmentation events are available for further branching and recombination. Our results indicate that the fragmentation of saturated molecules driven by high-energy processes, such as shocks, cosmic rays, cosmic-ray-induced UV fields, and X-rays, provides a viable pathway for the formation of unsaturated species, including the highly unsaturated carbon chains observed in regions like G+0.693-0.027. These processes naturally favor the production of fragments with the maximum possible number of π bonds, which are stabilized by electron delocalization and characterized by minimal hydrogen content. This work highlights the pivotal role of high-energy conditions in shaping the chemical complexity of molecular clouds and offers a promising framework for investigating the formation and detection of complex molecular fragments in the ISM.

## Data availability

The data supporting this article have been included as part of the ESI.†

## Author contributions

Conceptualization: JL-R, SG, HMQ-L, FF, AR; data curation: JL-R, SG; formal analysis: JL-R, SG, AR; funding acquisition: HMQ-L, FF, AR; investigation: JL-R, SG, HMQ-L, FF, AR; methodology: JL-R, SG, HMQ-L, FF, AR; project administration: HMQ-L, FF, AR; software: JL-R, SG; supervision: HMQ-L, FF, AR; validation: JL-R, SG, HMQ-L, FF, AR; visualization: JL-R, SG, FF; writing original draft: JL-R, SG, HMQ-L, FF, AR; writing reviewing and editing: JL-R, SG, HMQ-L, FF, AR.

## Conflicts of interest

There are no conflicting interests to declare.

## Supplementary Material

SC-016-D4SC07986H-s001
