# Supplementary material for: More π, please: What drives the formation of unsaturated molecules in the interstellar medium?
Source: Chem Sci. 2025 Jan 2;16(7):3051–65. doi: 10.1039/d4sc07986h (PMC11718301; doi:10.1039/d4sc07986h)
Supplement: SC-016-D4SC07986H-s001 [file SC-016-D4SC07986H-s001.pdf]

Supplementary Information (SI) for Chemical Science.
This journal is © The Royal Society of Chemistry 2025

Supplementary Information for

More π, please: What drives the formation of unsaturated molecules in the interstellar medium?

Jhoan Londoño-Restrepo,[a‡] Santiago Gómez,[b,c‡] Heidy M. Quitián-Lara,*[d,e] Felipe Fantuzzi*[f] and Albeiro Restrepo*[b]

[a] *Instituto de Física, Universidad de Antioquia, Calle 70 No. 52-21, Medellín, Colombia.*

[b] *Instituto de Química, Universidad de Antioquia, Calle 70 No. 52-21, Medellín 050010, Colombia. E-mail: albeiro.restrepo@udea.edu.co.*

[c] *Department of Physics, Chemistry and Biology (IFM), Linköping University, Linköping SE-581 83, Sweden.*

[d] *Max Planck Institute for Extraterrestrial Physics, Gießenbachstraße 1, 85748 Garching, Germany. E-mail: heidyql@mpe.mpg.de.*

[e] *Physics and Astronomy, School of Engineering, Mathematics and Physics, University of Kent, Park Wood Rd, Canterbury CT2 7NH, UK.*

[f] *Chemistry and Forensic Science, School of Natural Sciences, University of Kent, Park Wood Rd, Canterbury CT2 7NH, UK. E-mail: f.fantuzzi@kent.ac.uk.*

‡ These authors contributed equally to this work.

Contents

S1 Main fragmentation paths **S3**

S2 Electronic activity **S13**

S3 Rate constants for the $m_1 \rightarrow m_2$ reactions **S15**

S4 Low-lying excited states of ionized GlA **S16**

S5 Stability of the highly unsaturated fragments of m/q = 64 **S19**

S6 Cartesian coordinates of the reported molecular fragments **S20**

S1 Main fragmentation paths

Ethanolamine (C_2H_7NO)

The three main fragmentation routes for ethanolamine obtained from the BOMD calculations, corresponding to the initial breaking of the C2–C3, C3–O4, and N1–C2 bonds, are shown in Figure S1. The most intense experimental peak in the mass spectrum (Figure 2A in the main text) with $m/q = 30$ matches the largest number of BOMD counts, which corresponds to $[H_2CNH_2]^+$. The rupture of the C2–C3 bond in ethanolamine leads to either $[H_2CNH_2]^+$ ($m/q = 30$) or $[H_2COH]^+$ ($m/q = 31$). The fewer counts for $[H_2COH]^+$ from the dynamics indicate that the positive charge is heavily favored to reside on the N-containing fragment, which also contributes to the $m/q = 31$ peak by capturing an additional H atom.

The central cluster of peaks in both the experimental and calculated spectra shows intense signals at $m/q = 28, 29$, which correspond to the second and third fragmentation events (sequential hydrogen loss leading to the highly unsaturated $[HCNH]^+$ and then to $[HCN]^+$ with $m/q = 27$) following the rupture of the C2–C3 bond, as shown in Figure S1. An additional structure with $m/q = 29$ results from $[HCOH]^+$ losing a hydrogen atom to become $[HCO]^+$. Notably, $[HCNH]^+$ and $[HCO]^+$ have been detected in Sgr B2 [1, 2] and are thus strong candidates to be found in G+ as well. Our results indicate that both $[H_2CNH_2]^+$ and $[H_2COH]^+$ are prime candidates to be found in G+ along with all the molecular ions in the $m/q = 27 - 31$ range.

The group of peaks with signals of $m/q = 41 - 43$ was obtained in both the experimental and calculated spectra. The neutral form of $[H_2CCHNH_2]^+$ ($m/q = 43$) is the only species so far detected along with ethanolamine in G+, suggesting that the fragmentation of ethanolamine may be a viable source of this fragment. [3] Notice that the $m/q = 42$ peak is the most intense in this group. Although neutral H_2CCO has been detected in Sgr B2, [4] the high number of counts in Table 1 in the main text suggests that other species with this mass to charge ratio, especially $[H_2CCNH_2]^+$ ($m/q = 42$), should also be considered.

The loss of an additional hydrogen atom from either the nitrogen or oxygen ends of the $m/q = 42$ fragments leads to the signal at $m/q = 41$, composed of the counts from three fragments, out of which $HNCCH_2$ has been detected in Sgr B2. [5] It is worth mentioning that the peak at $m/q = 61$ corresponds to cationic ethanolamine, for which the BOMD calculations did not show any further rearrangements. The peak at $m/q = 60$ is heavily populated with at least six isomers, as listed in Figure S2. The peak at $m/q = 18$, most likely corresponding to water and protonated ammonia, is also quite intense in the experiments but only marginally produced in our calculations given the time span considered in the BOMD.

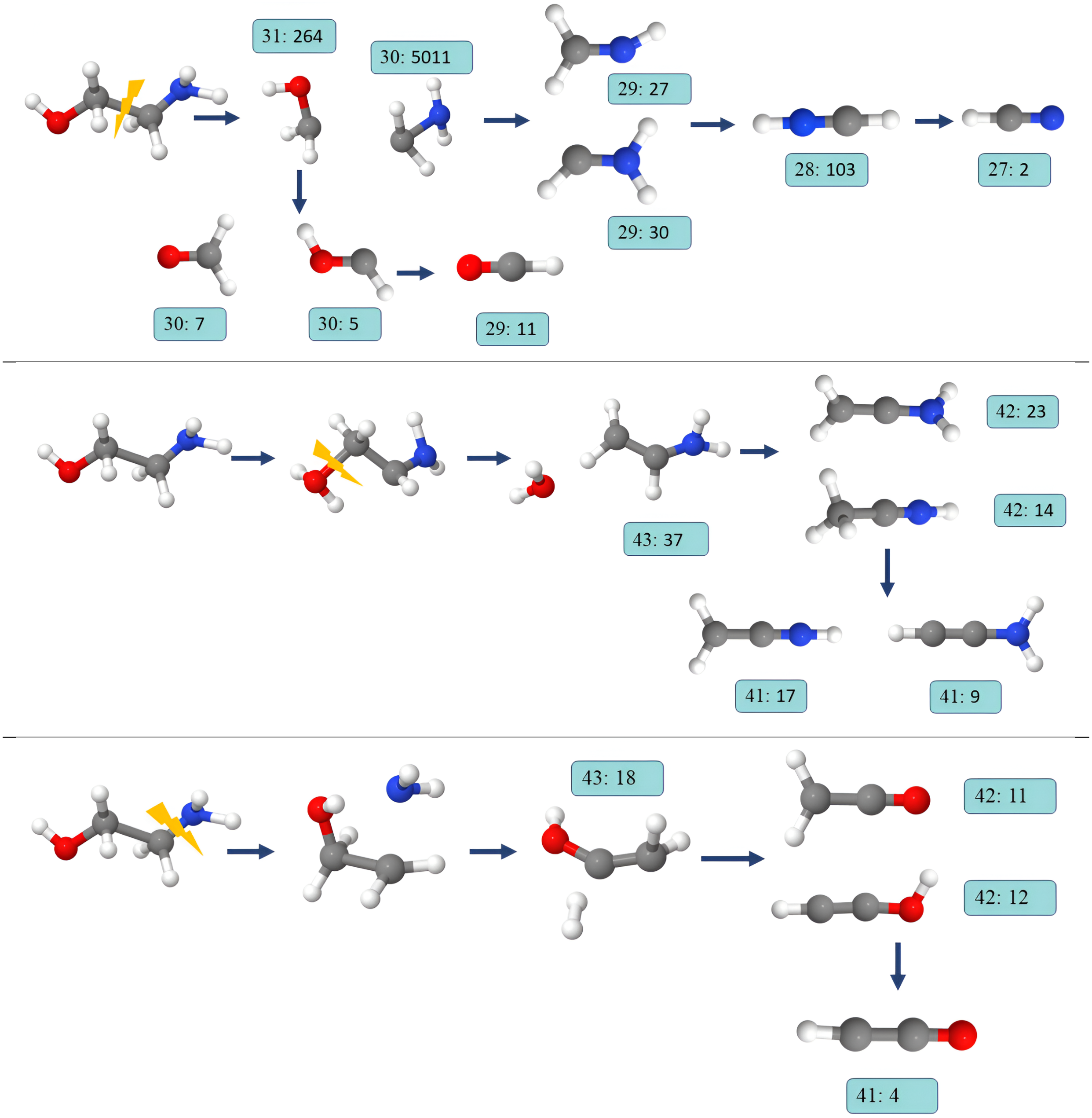

Figure S1: The three main fragmentation routes of ethanolamine: rupture of the C–C, C–O, and C–N bonds are shown at the top, middle, and bottom panels, respectively. Notice that these are the structures taken from the snapshots of the BOMD simulations. The cartesian coordinates of the actual optimized geometries are given in section S6. The boxed numbers $\boxed{xx : yy}$ indicate the m/q ratio and the number of counts for each fragment in the 100 ps dynamics.

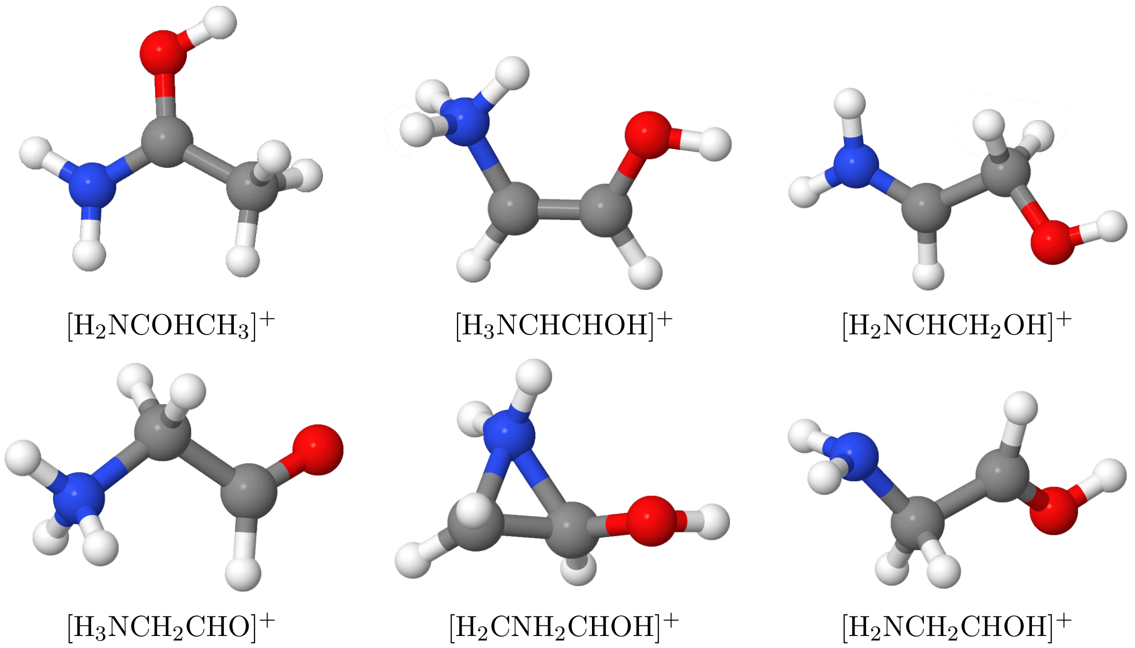

Figure S2: Optimized structures of the six distinct isomers with $m/q = 60$ produced from ethanolamine.

Propanol

The BOMD simulations identified three main fragmentation routes for propanol, corresponding to the breaking of the interior C2–C3 bond, the terminal C1–C2 bond, and the C3–O4 bond. These routes are schematized in Figure S3. An alternative route involving the rupture of a C–H bond adjacent to the OH group, leading to a fragment of $m/q = 59$, is also provided.

Breaking the interior C2–C3 bond leads to the two most intense peaks in the experimental and computed spectra (Figure 2 in the main text): one peak corresponding to the $[H_2CHCH_2]^+$ fragment with $m/q = 29$, and one peak corresponding to the $[H_2COH]^+$ fragment with $m/q = 31$. Once the $[H_2CHCH_2]^+$ and $[H_2COH]^+$ fragments are produced, they proceed to lose hydrogen atoms, subsequently yielding the highly unsaturated $[HCCH]^+$ ($m/q = 26$) and $[HCO]^+$ ($m/q = 29$) fragments, respectively.

The breakage of the C3–O4 bond produces the second fragmentation path for propanol. This path is the major contributor to the cluster of peaks centered around $m/q = 40$. The main product of this fragmentation is $[H_2CCHCH_3]^+$, which proceeds to lose hydrogen atoms, ultimately producing the highly unsaturated $[HCCCH_2]^+$ ($m/q = 39$) and eventually $[HCCCH]^+$ ($m/q = 38$).

The third fragmentation route for propanol begins with the rupture of the terminal C1–C2 bond. Subsequently, the oxygenated fragment bifurcates into two different paths that finally converge on the $m/q = 43$ $[H_2CCOH]^+$ fragment. This fragment, by losing two different hydrogen atoms, leads to two $m/q = 42$ species: $[HCCOH]^+$ and $[H_2CCO]^+$. Notably, the $m/q = 42$ peak is quite intense because it has contributions from three different structures.

Notice that $[HCOH]^+$, $[H_2COH]^+$, and $[H_2CCO]^+$, which are fragments common to the fragmentation paths of both ethanolamine and propanol, have already been detected in Sgr B2. Thus, $[H_2CCOH]^+$, $[HCCOH]^+$, $[H_2CCO]^+$, $[H_2CO]^+$, and $[HCO]^+$, which are also common to the fragmentation of ethanolamine and propanol, should be prime candidates for astrophysical detection. In addition, $[H_2CHCH_2]^+$ and $[H_2CCHCH_3]^+$, as well as their derivatives after losing hydrogen atoms, are also good candidates for detection.

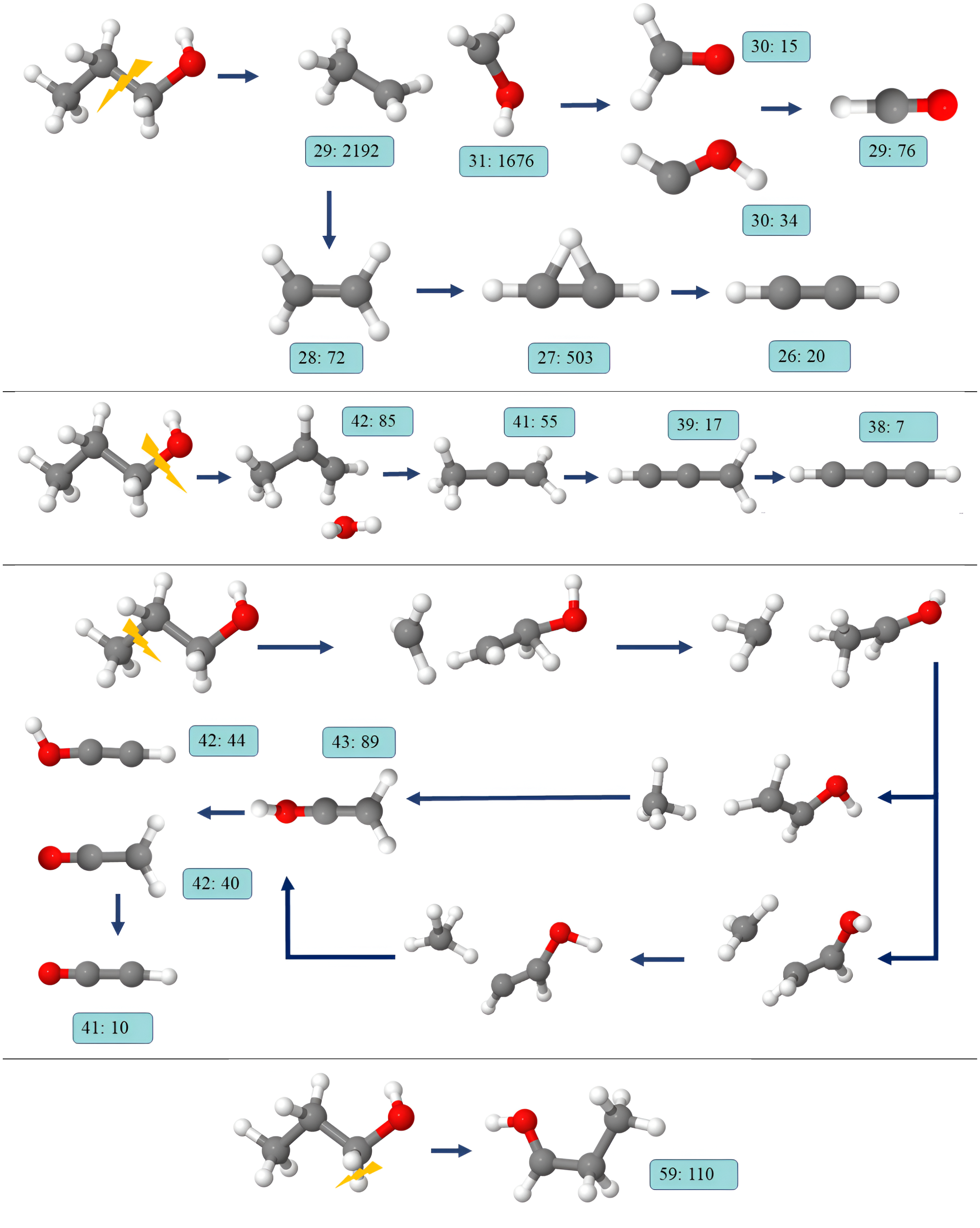

Figure S3: The three main fragmentation routes of propanol: rupture of the C2–C3, C3–O4, and C1–C2 bonds are shown at the top, second, and third panels, respectively. An additional route involving the breaking of one C–H bond adjacent to the OH group is shown at the bottom. Notice that these are the structures taken from the snapshots of the BOMD simulations. The cartesian coordinates of the actual optimized geometries are given in section S6. The boxed numbers $\boxed{xx : yy}$ indicate the m/q ratio and the number of counts for each fragment in the 100 ps dynamics.

Butanenitrile

The BOMD simulations produce three radical structural isomers when butanenitrile is ionized: (i) $[H_2CCH_2CH_2CNH]^+$, the lowest energy isomer, which we refer to as **BTN3**; (ii) $[H_3CCHCH_2CHN]^+$, the second lowest energy structure, which we call **BTN2**; and (iii) $[H_3CCH_2CH_2CN]^+$, the cation with the same connectivity among the atoms as in the neutral parent, which we call **BTN1**. Several competitive processes follow: structural isomerization **BTN1** $\rightarrow$ **BTN2** and **BTN1** $\rightarrow$ **BTN3**, in addition to fragmentation of each cation. The most important fragmentation patterns for these three cations obtained from the simulations are provided in Figure S4.

SI/fragmentation of BTN1. There are two fragmentation routes for this isomer, involving the rupture of either the central C2–C3 bond (top row in Figure S4) or the terminal C1–C2 bond (second row in Figure S4). Breaking the central C2–C3 bond produces two different cations: $[H_2CCN]^+$ with m/q=40, which eventually loses one hydrogen atom to become $[HCCN]^+$, and $[H_2CHCH_2]^+$ with $m/q = 29$, the cation with the largest number of counts by far. The $[H_2CHCH_2]^+$ fragment continues to lose hydrogens until it finally yields the highly unsaturated $[HCCH]^+$.

Breaking the terminal C1–C2 bond yields either $[CH_3]^+$ ($m/q = 15$) or $[H_2CCH_2CN]^+$ ($m/q = 54$). This latter fragment undergoes very rapid reorganization to produce two structural isomers, which proceed to lose hydrogens, ultimately yielding the highly unsaturated $[HCCCNH]^+$, $[H_2CCCN]^+$, $[HCHCCN]^+$ (cyclic) and $[HCCCN]^+$ fragments, which are noticeable in the experimental spectrum.

SI/fragmentation of BTN2. The third row in Figure S4 shows the structural isomerization of **BTN1** into **BTN2** and its subsequent fragmentation after the breaking of the C3–C4 bond. An alternative route involves the loss of H_2 from **BTN1** , followed by fragmentation after breaking the same C3–C4 bond. An interesting observation is that, regardless of the location of the two hydrogens lost as H_2 from **BTN1**, the structure about to break the C3–C4 bond, as shown in the third row of Figure S4, is always obtained after structural reorganization. These two routes converge to $[H_3CCCH_2]^+$ ($m/q = 41$) which, like all other fragmentation patterns, continues to increase its unsaturation by losing hydrogens until finally arriving at $[HCCCH]^+$.

SI/fragmentation of BTN3. The bottom row of Figure S4 shows the fragmentation of **BTN3** following the structural reorganization of **BTN1**. According to the BOMD simulations, the rupture of the central C2–C3 bond occurs so rapidly after the structural isomerization of **BTN1** that it prevents alternative fragmentation paths via the rupture of the other two C−C bonds in **BTN3**. Breaking the central C2–C3 bond produces either $[H_2CCH_2]^+$, a fragment with $m/q = 28$ that is common to one of the fragmentation routes of **BTN1** and eventually yields $[HCCH]^+$, or $[H_2CCNH]^+$, a fragment with $m/q = 41$ which leads to unsaturated fragments with $m/q = 40$ and $m/q = 39$, also formed from the fragmentation of **BTN1**.

As mentioned earlier, neutral butanenitrile has been detected in Sgr B2. [6] Additionally, neutral H_2CCN, H_3CCCH, H_2CCNH, H_3CCN and HCCCN fragments have also been detected. [5,7–10] There are several fragments that are common to the fragmentation routes of ethanolamine and propanol, including the two (undetected yet) fragments with the highest counts: $[H_2CHCH_2]^+$ with $m/q = 29$, and $[HCCHCH]^+$ with $m/q = 27$. These fragments have unusual cyclic structures where the central hydrogen atom bridges two carbons. All the undetected fragments are good candidates to be found either in G+ or in Sgr B2.

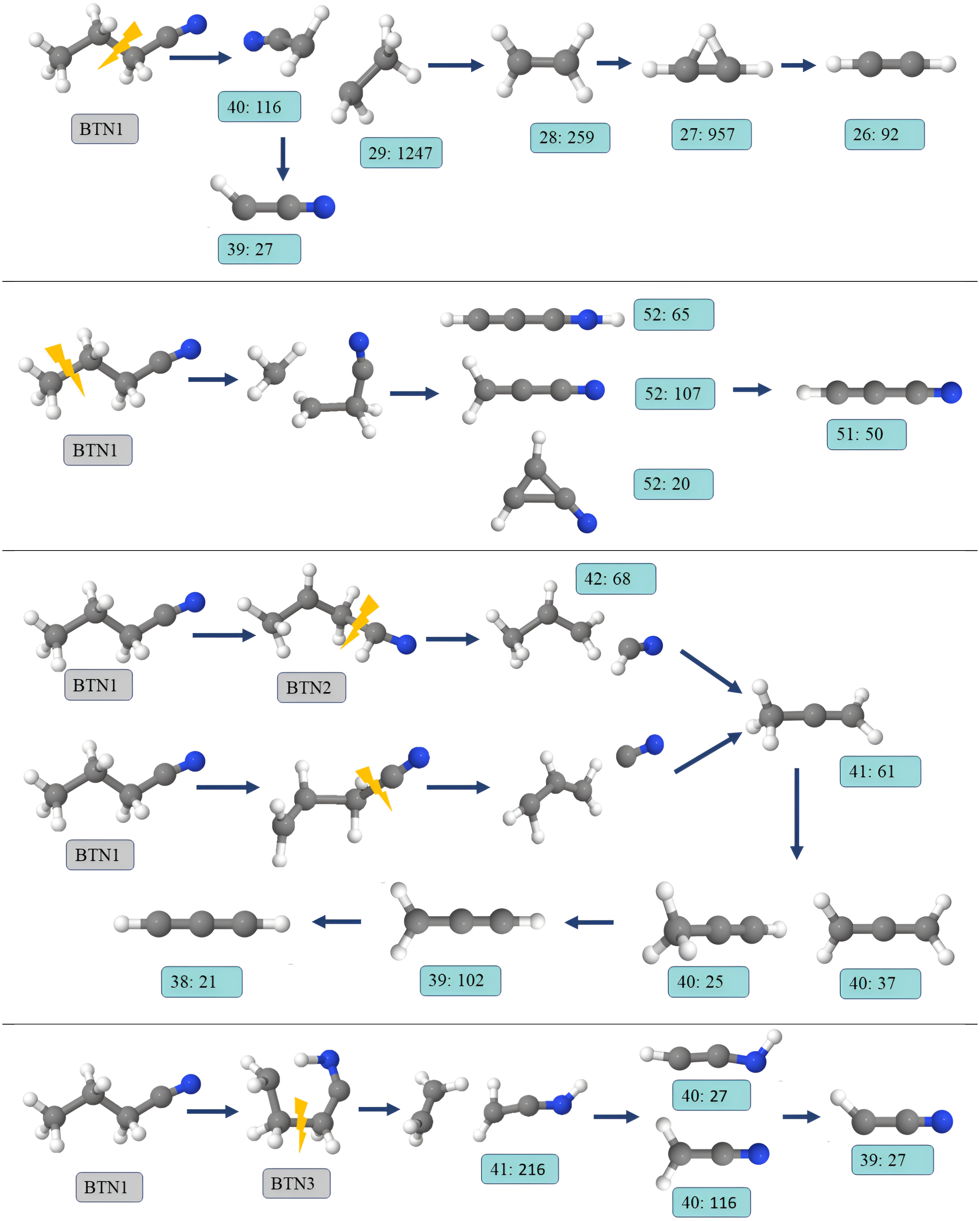

Figure S4: Main fragmentation routes of butanenitrile for the rupture of the C–C bonds. Notice that these are the structures taken from the snapshots of the BOMD simulations. The cartesian coordinates of the actual optimized geometries are given in section S6. The boxed numbers $\boxed{xx : yy}$ indicate the m/q ratio and the number of counts for each fragment in the 100 ps dynamics.

Glycolamide

Three fragmentation routes for glycolamide involving the rupture of the C2–C3 bond are identified by the BOMD simulations, as shown in Figure S5.

The first fragmentation route produces either one of two peaks: the most intense signal in both the experimental and computed spectra, which corresponds to $[H_2NCO]^+$ with $m/q = 44$, and the second most intense peak, corresponding to $[H_2COH]^+$ with $m/q = 31$. Both cations have been observed in Sgr B2. [11, 12]

The second fragmentation produces cationic methanol ($[CH_3OH]^+$ with $m/q = 32$, which has been observed in its neutral form in Sgr B2. [13] Finally, the last fragmentation involves an intramolecular proton transfer followed by the rupture of the C2–C3 bond, producing the $[H_2NCOH]^+$ fragment with $m/q = 45$.

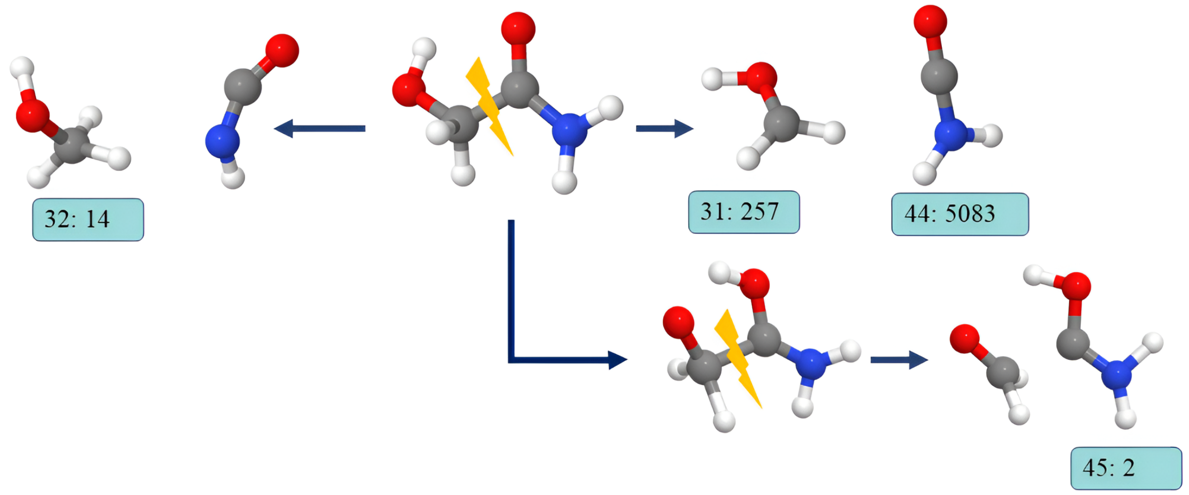

Figure S5: Main fragmentation routes of glycolamide for the rupture of the C–C bond. Notice that these are the structures taken from the snapshots of the BOMD simulations. The cartesian coordinates of the actual optimized geometries are given in section S6. The boxed numbers $\boxed{xx : yy}$ indicate the m/q ratio and the number of counts for each fragment in the 100 ps dynamics.

Table S1: List of most important fragments featuring a single π bond.

m/q	Molecular Formula	Species
26	C_2H_2	$[HCCH]^+$
27	C_2H_3	$[HCHCH]^+$
27	CHN	$[HCN]^+$
28	C_2H_4	$[H_2CCH_2]^+$
28	CH_2N	$[HCNH]^+$
28	CH_2N	$[H_2CN]^+$
29	CH_3N	$[HCNH_2]^+$
29	CH_3N	$[H_2CNH]^+$
29	CHO	$[HCO]^+$
30	CH_2O	$[H_2CO]^+$
30	CH_2O	$[HCOH]^+$
30	CH_4N	$[H_2CNH_2]^+$
31	CH_3O	$[H_2COH]^+$
40	C_3H_4	$[H_3CCCH]^+$
41	C_2H_3N	$[H_3CCN]^+$
41	C_3H_5	$[H_3CCCH_2]^+$
42	C_2H_2O	$[HCCOH]^+$
42	C_2H_4N	$[H_3CCNH]^+$
43	C_2H_3O	$[H_3CCO]^+$
45	CH_3NO	$[H_2NCOH]^+$
59	C_3H_7O	$[H_3CCH_2CHOH]^+$
60	C_2H_6NO	$[H_2NCOHCH_3]^+$
60	C_2H_6NO	$[H_3NCHCHOH]^+$
60	C_2H_6NO	$[H_2NCHCH_2OH]^+$
60	C_2H_6NO	$[H_3NCH_2CHO]^+$
60	C_2H_6NO	$[H_2NCH_2CHOH]^+$

Table S2: List of most important fragments featuring multiple π bonds.

m/q	Molecular Formula	Species
38	C_3H_2	$[HCCCH]^+$
39	C_3H_3	$[H_2CCCH]^+$
39	C_2HN	$[HCCN]^+$
40	C_2H_2N	$[H_2CCN]^+$
40	C_2H_2N	$[HCCNH]^+$
40	C_3H_4	$[H_2CCCH_2]^+$
41	C_2H_3N	$[H_2CCNH]^+$
41	C_2H_3N	$[HCCNH_2]^+$
41	C_2H_3N	$[H_2CNCH]^+$
41	C_2HO	$[HCCO]^+$
42	C_2H_2O	$[H_2CCO]^+$
42	C_2H_4N	$[H_2CCNH_2]^+$
43	C_2H_3O	$[H_2CCOH]^+$
44	CH_2NO	$[H_2NCO]^+$
51	C_3HN	$[HCCCN]^+$
52	C_3H_2N	$[HCCCNH]^+$
52	C_3H_2N	$[HCNCCH]^+$
52	C_3H_2N	$[H_2CCCN]^+$
52	C_3H_2N	$[c\text{-}HCCHCN]^+$
64	C_4H_2N	$[H_2CCCCN]^+$
64	C_4H_2N	$[HCCCHCN]^+$
64	C_4H_2N	$[HCCCCNH]^+$

S2 Electronic activity

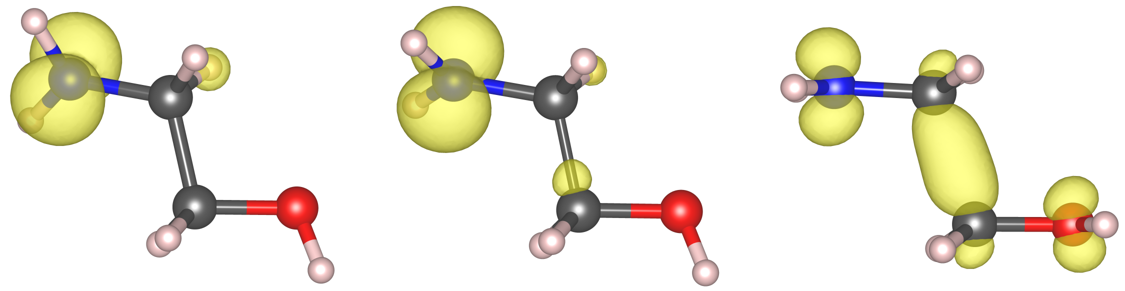

$m1$, $\mathrm{WBI_{C\text{-}C}} = 0.98$ TS, $\mathrm{WBI_{C\text{-}C}} = 0.95$ $m2$, $\mathrm{WBI_{C\text{-}C}} = 0.58$

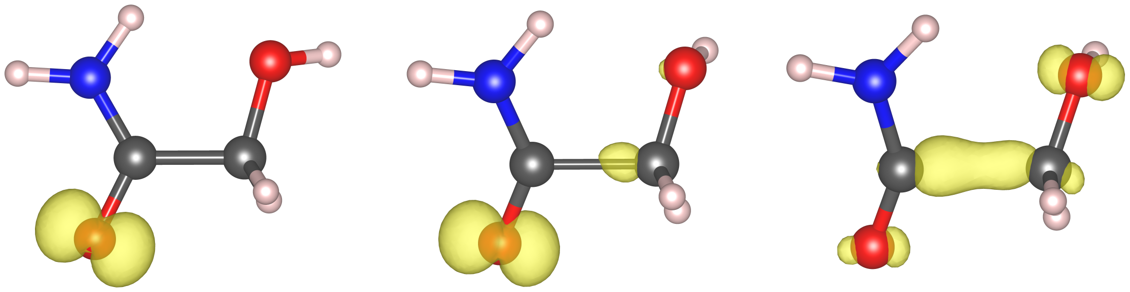

$m1$, $\mathrm{WBI_{C\text{-}C}} = 0.97$ TS, $\mathrm{WBI_{C\text{-}C}} = 0.79$ $m2$, $\mathrm{WBI_{C\text{-}C}} = 0.49$

Figure S6: Electronic activity for the $m1 \to \mathrm{TS} \to m2$ process involved in the rupture of the C–C bond in ethanolamine (top) and glycolamide (bottom). The gradual increase of the α spin density in the central C–C bond by virtue of the transfer of the β electron to finally obtain a 2c–1e C–C bond is clearly seen. WBI stands for the Wiberg Bond Index.

Table S3: Number of electrons (N_e) and Wiberg Bond Indices (WBI) for the C–C bonds in the predissociative complexes.

Molecule	Basin	Basin type	Integral of Electron Density (N_e)	Bond order (WBI)	AdNDP population (N_e)
Ethanolamine (EtA)	V(C2,C3)	disynaptic	0.95	0.58	0.99
Propanol (PrO)	V(C2,C3)	disynaptic	1.08	0.62	0.99
Butanenitrile (BuN)	V(C2,C3)	disynaptic	1.03	0.67	0.97
Glycolamide (GlA)	V(C2),V(C3)	monosynaptic	0.42, 0.63	0.49	0.98

S3 Rate constants for the $m_1 \rightarrow m_2$ reactions

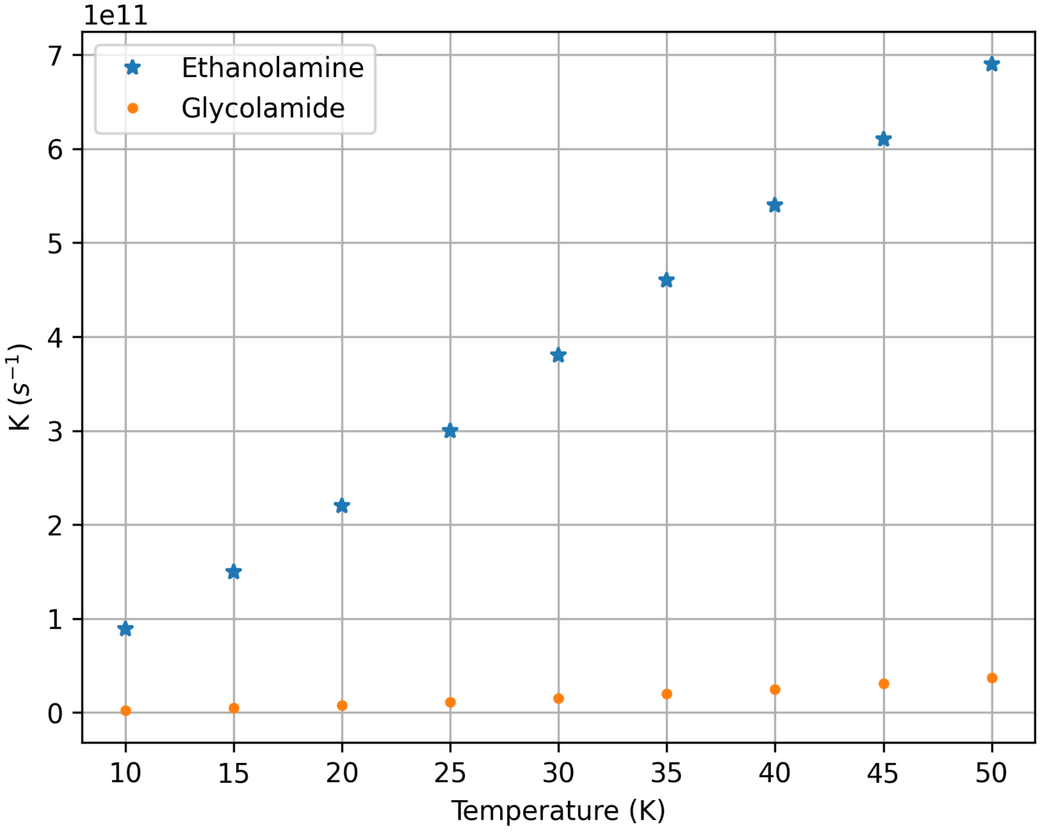

Figure S7: Rate constants for the $m_1 \rightarrow m_2$ reactions leading to the cleavage of the C–C bond in Ethanolamine and Glycolamide as a function of temperature. All constants were calculated with the EyringPy 2.0 program [14] using Eckart tunneling corrections.

S4 Low-lying excited states of ionized GlA

Figure S8 presents a bar plot comparing energy gaps between the D_0 and D_1 states at selected points along the potential energy curve of the C2−C3 dissociation of ionized **GlA**. These energy gaps were computed at the state-averaged complete active space self-consistent field (SA-CASSCF) and the resolution-of-the-identity N-electron valence state second-order perturbation theory (RI-NEVPT2) levels. The selected points correspond to key geometries along the dissociation pathway: 1.30 Å, before the first minimum on the MP2 potential energy surface; 1.54 Å, at the first MP2 minimum; 1.70 Å, at the maximum between the two MP2 minima; 1.98 Å, at the elongated MP2 minimum; and 2.30 Å, beyond the elongated MP2 minimum.

For the SA-CASSCF calculations, two distinct active spaces were employed to systematically evaluate the impact of active space size on the computed energy gaps: a smaller active space with 3 electrons in 4 orbitals (3,4) and a larger active space with 11 electrons in 12 orbitals (11,12). The calculations using the smaller active space were performed with the triple-ζ def2-TZVP basis set, while the larger active space calculations utilized the double-ζ def2-SVP basis set. To provide a more accurate description of the electronic structure, dynamic correlation effects were incorporated for both active spaces using RI-NEVPT2.

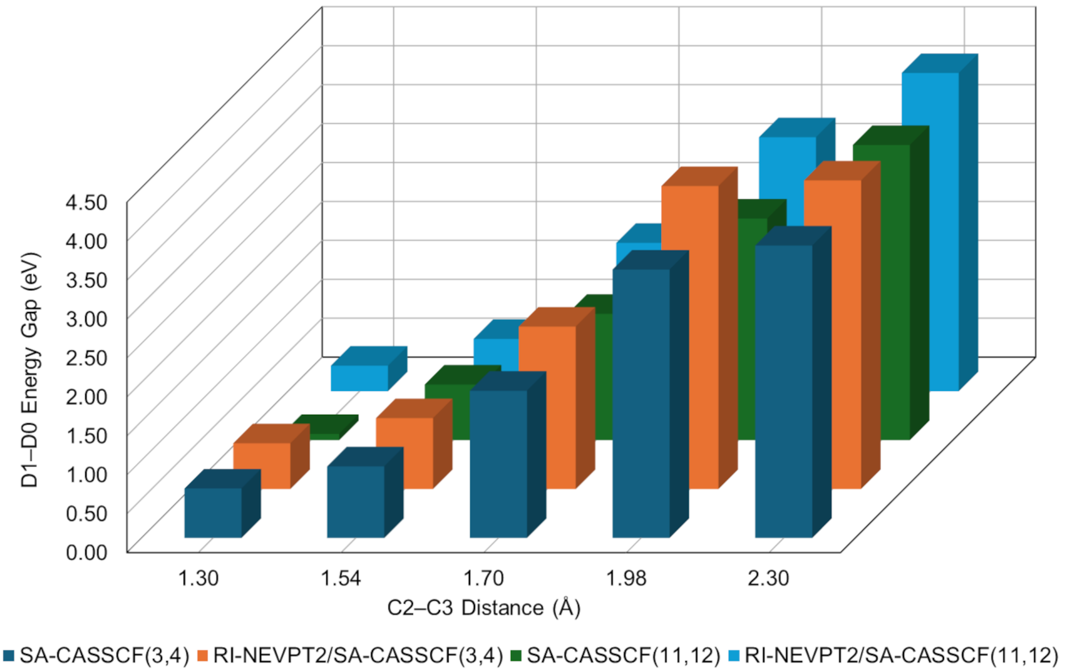

Figure S8: Comparison of energy gaps between the D_0 and D_1 states at selected geometries along the C2–C3 dissociation pathway of ionized **GlA**, as computed at the SA-CASSCF and RI-NEVPT2 levels. Results are shown for two active spaces: 3 electrons in 4 orbitals (3,4) and 11 electrons in 12 orbitals (11,12). The selected geometries correspond to key points in the MP2-optimized potential energy curve. See the text for further discussion.

The results demonstrate that, irrespective of the computational method or the inclusion of dynamical correlation with RI-NEVPT2, the energy gap between D_0 and D_1 consistently increases as the C2−C3 bond lengthens. For example, at the SA-CASSCF(3,4) level, the energy gaps increase from 0.64 eV at 1.30 Å to 0.92 eV at 1.54 Å, 1.89 eV at 1.70 Å, 3.45 eV at 1.98 Å, and 3.76 eV at 2.30 Å. When dynamical correlation is included at the RI-NEVPT2 level, the

energy gaps similarly increase, albeit with slight variations: 0.59 eV, 0.91 eV, 1.90 eV, 3.45 eV, and 3.76 eV at the respective points. These results indicate that dynamical correlation slightly decreases the gap for shorter C2−C3 bond lengths but progressively amplifies it for elongated bond geometries, with an additional 0.45 eV at the elongated minimum relative to SA-CASSCF(3,4).

A similar trend is observed for the larger active-space SA-CASSCF(11,12) and its corresponding RI-NEVPT2 calculations. For these methods, the inclusion of dynamical correlation increases the energy gap at the elongated minimum by 0.42 eV compared to SA-CASSCF(11,12) alone. These consistent increases in the D_0–D_1 gap across all methods confirm that the maximum in the potential energy curve between the two minima is not caused by an avoided crossing. Instead, the observed behavior arises from structural reconfigurations of the surrounding substituents as the C2–C3 bond length increases.

The comparison of SA-CASSCF and RI-NEVPT2 results underscores an important observation: the trends in energy gaps between the D_0 and D_1 states can be reliably captured without the need for multireference calculations. To further validate this observation and simplify the computational framework, we computed the excited-state curves using time-dependent density functional theory (TDDFT) with the ωB97X-D3BJ functional. This functional was chosen for its demonstrated reliability in predicting excitation energies and charge-transfer states, making it well-suited for excited-state investigations.

Figure S9 compares the ground-state potential energy curve obtained using TDDFT (ωB97X-D3BJ) with the MP2 method along the C2–C3 dissociation pathway. Both methods reveal qualitatively similar features, including two distinct minima corresponding to the short and elongated C2–C3 bond configurations. The ωB97X-D3BJ functional successfully reproduces the overall shape of the potential energy curve. However, quantitative differences are observed. For instance, while MP2 predicts a deeper secondary minimum at the elongated bond, ωB97X-D3BJ suggests a less pronounced stabilization. Additionally, the position of the first minimum differs between the two methods: MP2 places it at 1.54 Å, while ωB97X-D3BJ predicts it at 1.70 Å, coinciding with the local maximum of the MP2 curve. Despite these deviations, the agreement between ωB97X-D3BJ and MP2 for the ground-state curve confirms that the TDDFT method is appropriate for examining the low-lying excited states of ionized **GlA**.

Figure S10 shows the potential energy curves of D_0 and the first three doublet excited states along the MP2-optimized C2–C3 dissociation pathway of ionized **GlA**. As indicated by the SA-CASSCF and RI-NEVPT2 results, the relative energy of the first doublet excited state (D_1) increases consistently with respect to D_0 as the dissociation progresses. At 1.70 Å, D_1 is located approximately 3.11 eV above D_0, rising monotonically to 5.38 eV at 2.30 Å. The higher excited states (D_2 and D_3) also increase in energy as the C2–C3 bond lengthens. These two states remain quasi-degenerate between 1.70 Å and 2.12 Å, after which D_2 decreases slightly and approaches the energy of D_1.

These findings reinforce the conclusions drawn from the SA-CASSCF and RI-NEVPT2 analyses: the low-lying excited states are sufficiently separated from D_0 to rule out the possibility of an avoided crossing along the dissociation pathway. Instead, the maximum observed in the MP2 potential energy curve is attributed to structural reconfigurations of the surrounding substituents as the C2–C3 bond length increases.

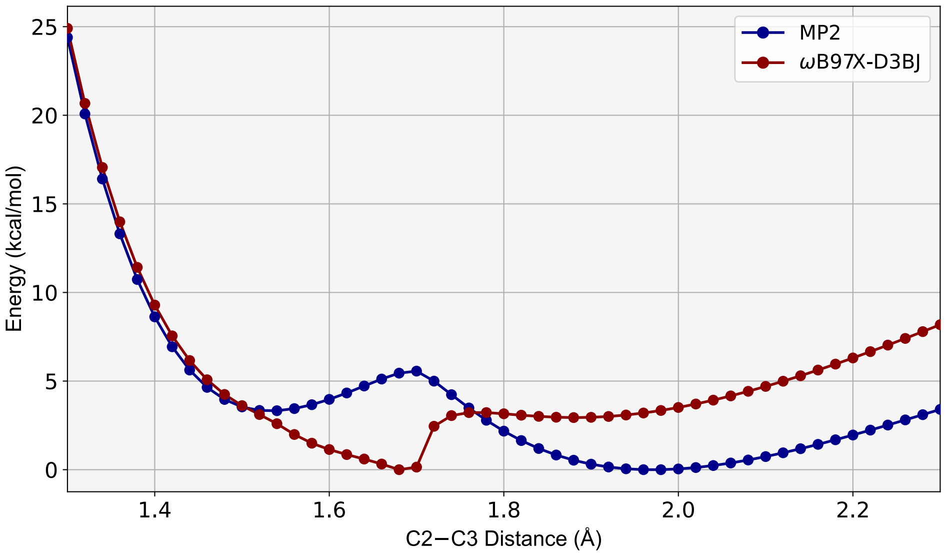

Figure S9: Comparison of the ground-state potential energy curves of ionized **GlA** obtained using TDDFT (ωB97X-D3BJ/def2-TZVP) and MP2 along the C2–C3 dissociation pathway.

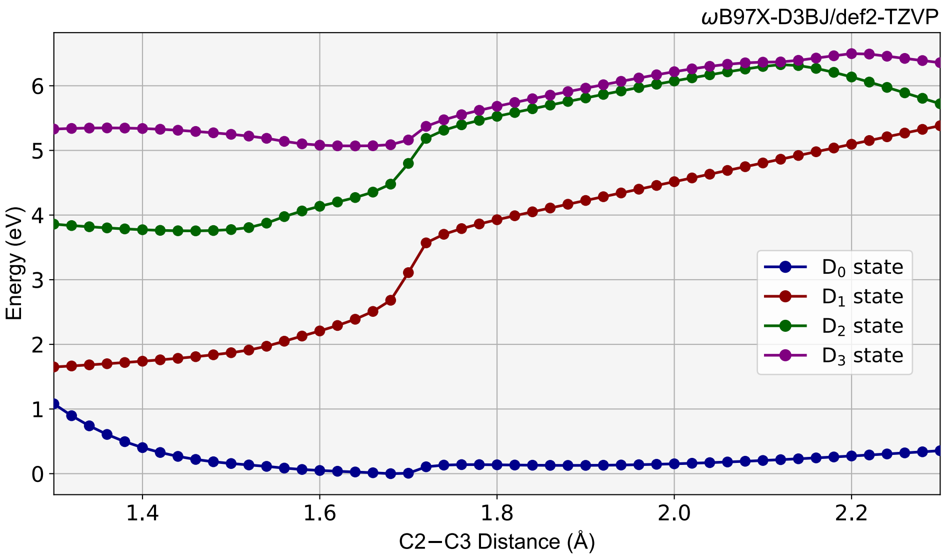

Figure S10: Potential energy curves of D_0 and the first three doublet excited states of ionized **GlA** (D_1–D_3) along the MP2-optimized C2–C3 dissociation pathway, as calculated using TDDFT (ωB97X-D3BJ/def2-TZVP).

S5 Stability of the highly unsaturated fragments of m/q = 64

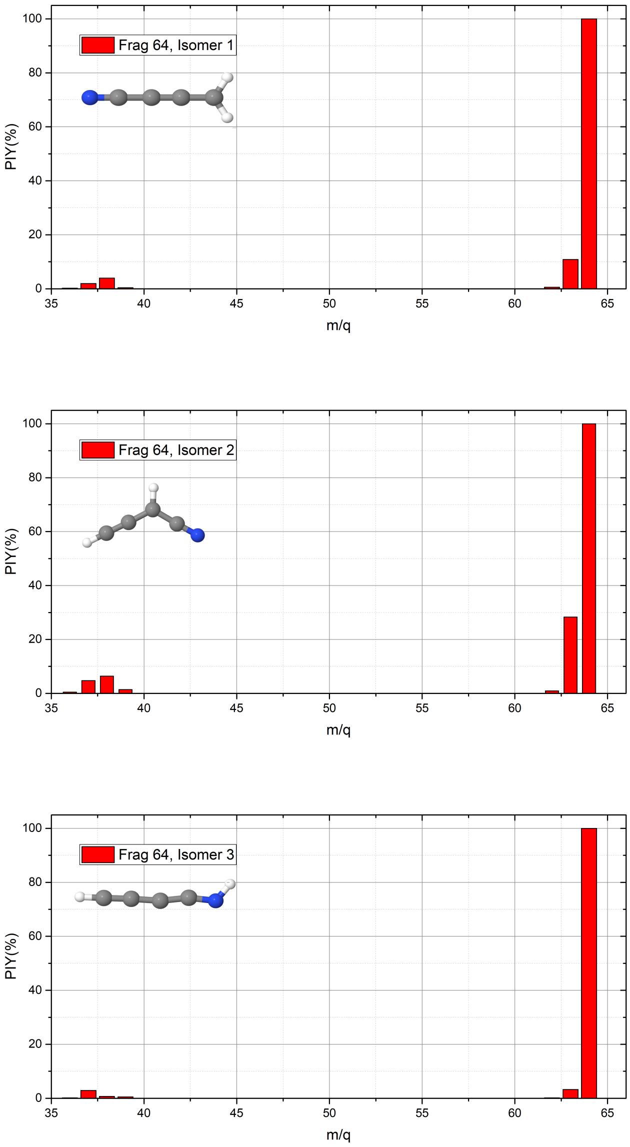

Figure S11: Calculated spectra for the $m/q = 64$ fragments of butanenitrile. See main text for further details.

S6 Cartesian coordinates of the reported molecular fragments

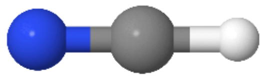

C	-1.195584	-35.780508	8.275950
N	-0.489304	-35.981620	9.230320
H	-1.842224	-35.600574	7.412080

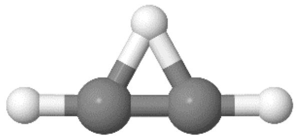

C	0.616741	-0.075752	-0.000094
C	-0.616723	-0.075707	-0.000007
H	1.696376	-0.069270	0.000399
H	-0.000117	1.047470	-0.000003
H	-1.696364	-0.069444	0.000210

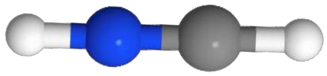

C	-48.664552	-11.218096	-9.165096
N	-48.312233	-11.505240	-10.197033
H	-47.998333	-11.762329	-11.126410
H	-49.001557	-10.943617	-8.173657

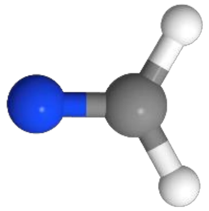

C	-1.225847	8.924434	0.230045
N	-0.293644	9.242158	0.905111
H	-1.678017	9.667049	-0.472317
H	-1.637359	7.888231	0.306567

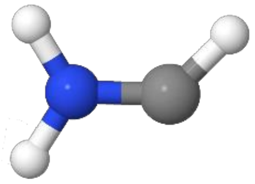

C	-14.643129	7.483539	-3.834682
N	-15.429246	7.469733	-4.804302
H	-15.453245	8.217295	-5.505396
H	-16.089116	6.700588	-4.939725
H	-13.875419	8.179683	-3.498661

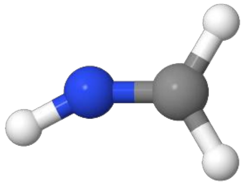

C	19.203181	0.859087	13.533672
N	19.656464	1.768387	12.851077
H	19.859988	2.202394	11.951670
H	18.738816	-0.019053	13.052962
H	19.254399	0.909551	14.630189

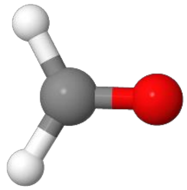

C	62.608645	171.445015	-101.852628
O	63.498366	171.943186	-101.246440
H	61.645885	171.212314	-101.336349
H	62.741364	171.213501	-102.937248

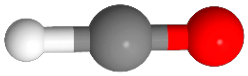

C	1.019761	-1.157990	-1.358373
O	0.059095	-0.918146	-0.881231
H	1.978547	-1.397121	-1.834899

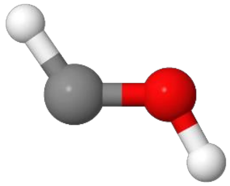

C	2.338344	-6.908111	-8.674730
O	2.318919	-5.751874	-8.309304
H	2.803369	-5.502485	-7.486055
H	1.833876	-7.293605	-9.575993

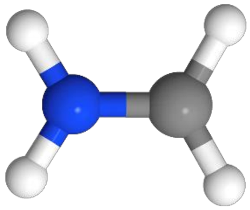

C	11.056524	-7.709599	17.386906
N	11.635963	-8.485473	18.206718
H	12.123160	-8.130855	19.024800
H	11.637063	-9.493409	18.078600
H	11.076020	-6.637587	17.555133
H	10.546455	-8.124772	16.523607

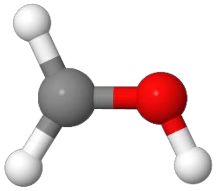

C	-12.533939	-15.239179	17.606451
O	-11.509524	-15.066785	18.293901
H	-11.408411	-15.354783	19.221639
H	-12.765643	-14.434753	16.904302
H	-13.139459	-16.152008	17.607417

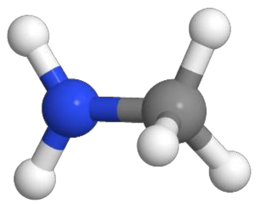

C	-17.688305	22.728798	-5.299112
N	-17.094913	23.852728	-4.691981
H	-16.294076	24.327413	-5.104841
H	-17.474307	24.252330	-3.835173
H	-18.004410	22.011467	-4.534261
H	-17.036168	22.294750	-6.052147
H	-18.627281	23.068614	-5.784016

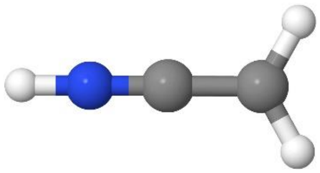

C	15.556504	-16.622921	27.288566
N	14.283694	-15.177158	28.918400
C	14.862463	-15.834078	28.179255
H	13.776773	-14.597928	29.570142
H	16.250305	-17.363879	27.668905
H	15.393974	-16.482927	26.225963

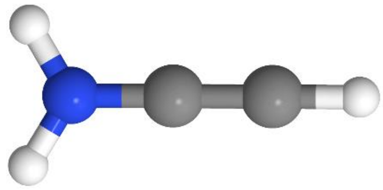

C	-13.920901	4.466592	-2.318340
N	-13.844641	4.424612	-3.592525
C	-13.992281	4.507099	-1.091024
H	-14.643714	4.666318	-4.173387
H	-12.982897	4.147683	-4.056303
H	-14.055470	4.543422	-0.020049

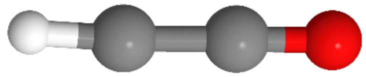

C	1.749738	-19.014830	20.229613
C	1.102220	-18.040928	21.002109
O	0.423783	-17.448136	21.671157
H	2.670497	-18.763100	19.689189

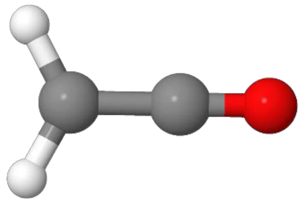

C	-5.910610	-5.361901	-2.162064
C	-5.535989	-4.049646	-1.961322
O	-5.234118	-2.984679	-1.798861
H	-6.912818	-5.664132	-1.868345
H	-5.190046	-6.046532	-2.602563

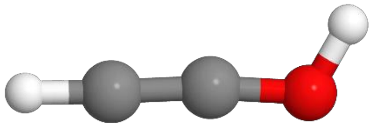

C	-2.921128	7.379907	12.074642
C	-4.044848	7.790818	12.384022
O	-5.197756	8.087558	12.709780
H	-5.415314	9.043867	12.704954
H	-1.935678	7.049674	11.798451

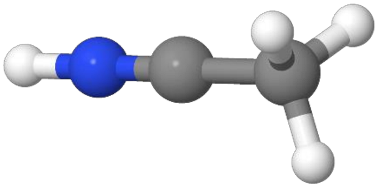

C	-2.539112	-5.684555	41.115765
N	-3.001764	-4.659195	41.269876
C	-1.954166	-6.981765	40.920054
H	-3.411279	-3.748093	41.406506
H	-1.175222	-7.133675	41.670623
H	-1.524317	-7.026144	39.916925
H	-2.733759	-7.738886	41.030680

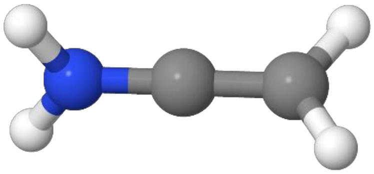

C	-6.552780	-6.858214	-4.478847
N	-6.220164	-5.784782	-5.034186
C	-6.895910	-7.951290	-3.914170
H	-5.480477	-5.199331	-4.652898
H	-7.676467	-7.950569	-3.158099
H	-6.676839	-5.467202	-5.886174
H	-6.408401	-8.883136	-4.187243

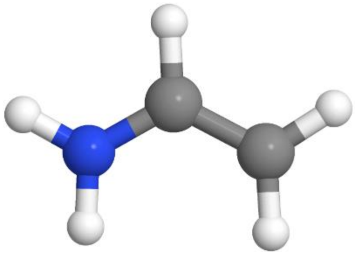

C	24.409832	0.572354	41.788299
N	25.411380	1.389378	41.598224
C	23.518157	0.704231	42.856955
H	25.594312	2.166969	42.221537
H	26.042486	1.271905	40.815705
H	22.706920	-0.001674	42.970115
H	23.621225	1.500443	43.585229
H	24.292056	-0.229521	41.067002

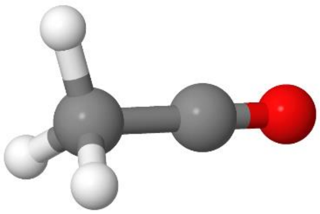

C	-1.223220	0.000001	-0.000073
C	0.215123	0.000175	0.000292
O	1.341298	-0.000054	-0.000093
H	-1.561029	-0.215482	1.019500
H	-1.559968	-0.775764	-0.696446
H	-1.560804	0.990625	-0.323629

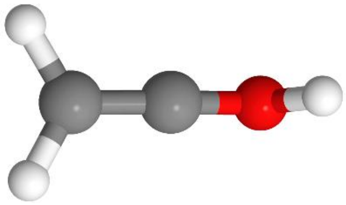

C	1.844676	-7.495388	-1.713225
C	2.058379	-7.480803	-0.461812
O	2.178634	-7.539962	0.750979
H	2.882873	-6.983868	1.148917
H	1.141411	-6.787190	-2.148497
H	2.380465	-8.200750	-2.346736

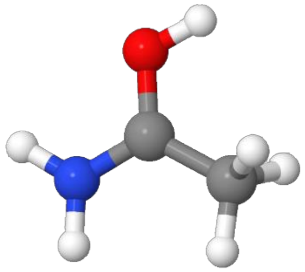

C	-11.935429	-1.800497	-0.390411
N	-11.527775	-3.161532	-2.373155
C	-11.140994	-2.747277	-1.207096
O	-10.010320	-3.219616	-0.789813
H	-12.394854	-2.844131	-2.776987
H	-10.960698	-3.812270	-2.901522
H	-9.752250	-2.890916	0.081238
H	-11.321825	-0.930164	-0.147716
H	-12.219950	-2.290145	0.543941
H	-12.833262	-1.471496	-0.907064

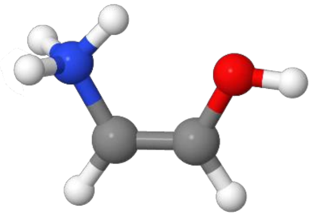

C	-14.586435	-8.760217	-5.432739
N	-13.943439	-8.178987	-6.619923
C	-14.125078	-8.401058	-4.242419
O	-13.110549	-7.535403	-4.151962
H	-14.592862	-7.614842	-7.172107
H	-12.855540	-7.354931	-3.243221
H	-13.176154	-7.569218	-6.318648
H	-14.560356	-8.802163	-3.334493
H	-15.396602	-9.446359	-5.611272
H	-13.552721	-8.896445	-7.234000

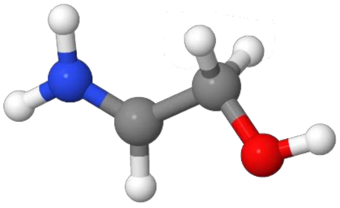

C	-0.245134	4.271702	-3.747491
N	-0.630062	3.444246	-4.641070
C	-0.949118	5.536754	-3.424061
O	-0.582774	5.870966	-2.126902
H	-0.094892	2.610398	-4.852950
H	-1.480282	3.587518	-5.175749
H	-0.795708	6.787437	-1.934611
H	-2.033166	5.415327	-3.557508
H	0.664656	4.044885	-3.195089
H	-0.604310	6.265379	-4.177836

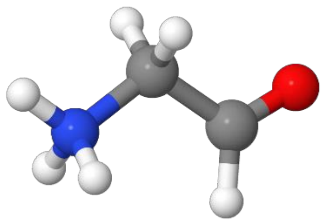

C	2.561623	-7.481753	-2.184136
N	2.126914	-6.118803	-2.650779
C	1.569413	-8.093491	-1.193167
O	1.738704	-9.196464	-0.790922
H	2.031583	-5.463547	-1.871760
H	1.227450	-6.154419	-3.135431
H	2.807019	-5.720662	-3.301239
H	0.709713	-7.465373	-0.875593
H	3.538396	-7.380559	-1.710570
H	2.656739	-8.125531	-3.058024

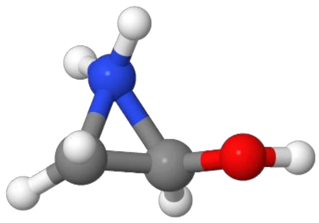

C	-7.376981	-4.767893	19.586275
N	-7.824695	-4.885066	21.002248
C	-8.805019	-4.969979	19.810536
O	-9.605097	-3.908647	19.672539
H	-7.580630	-5.715160	21.528586
H	-7.864875	-4.029088	21.544370
H	-10.518532	-4.109256	19.903335
H	-9.227724	-5.965194	19.716701
H	-6.762636	-5.589290	19.244273
H	-7.046433	-3.772843	19.316839

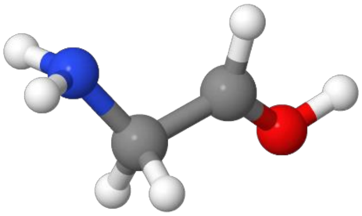

C	-7.236813	-12.602324	5.590664
N	-7.557922	-12.533343	4.197438
C	-6.741276	-11.295198	6.048220
O	-6.006886	-11.208456	7.065418
H	-8.432036	-12.974509	3.960148
H	-6.826257	-12.884966	3.599051
H	-5.747456	-10.302878	7.311092
H	-6.543354	-13.389964	5.913580
H	-7.019257	-10.396410	5.489326
H	-8.141935	-12.756230	6.204935

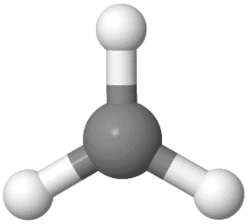

C	-0.000044	-0.000035	0.000022
H	0.815310	-0.724918	-0.000045
H	0.220501	1.068380	-0.000045
H	-1.035548	-0.343252	-0.000045

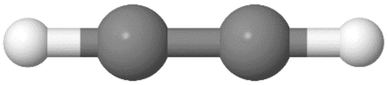

C	-0.628540	0.000074	-0.000002
C	0.628562	-0.000351	0.000001
H	-1.708499	0.000363	0.000007
H	1.708370	0.001301	0.000000

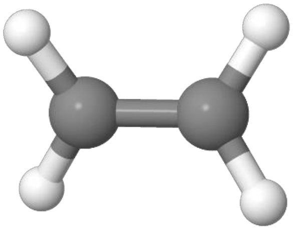

C	0.708094	-0.000001	0.000001
C	-0.708090	0.000000	0.000001
H	1.260347	0.932491	0.085964
H	-1.260365	-0.932481	0.085965
H	-1.260353	0.932487	-0.085972
H	1.260349	-0.932489	-0.085971

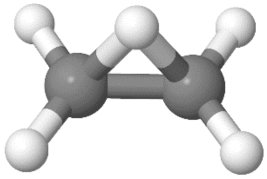

C	-0.692680	-0.000006	-0.064221
C	0.692768	-0.000030	-0.064219
H	-1.244101	-0.937370	-0.069484
H	-1.244050	0.937262	-0.069712
H	1.243780	-0.937623	-0.069548
H	1.243739	0.937586	-0.069747
H	0.000104	0.000359	1.049130

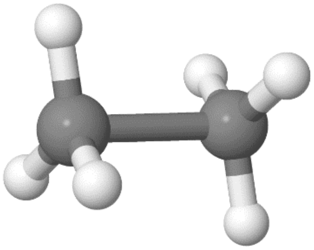

C	0.000000	0.000000	0.953304
H	0.000000	-1.075436	1.111341
H	0.931355	0.537718	1.111341
H	-0.931355	0.537718	1.111341
C	0.000000	0.000000	-0.953304
H	0.931355	-0.537718	-1.111341
H	-0.931355	-0.537718	-1.111341
H	0.000000	1.075436	-1.111341

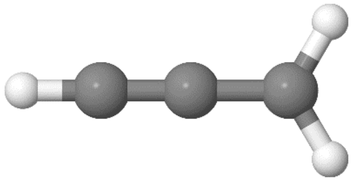

C	-0.108608	-0.000061	0.000146
C	1.249070	0.000021	-0.000087
C	-1.339007	0.000004	-0.000042
H	-2.415064	0.000049	-0.000141
H	1.802984	0.940140	0.000021
H	1.803349	-0.939975	0.000021

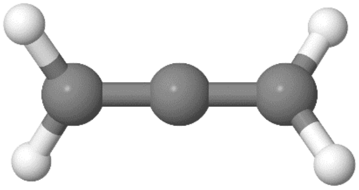

C	0.000000	0.000000	0.000000
C	1.311622	0.000000	-0.000149
C	-1.311623	0.000000	0.000149
H	1.868885	0.848190	-0.403516
H	1.868979	-0.848189	0.403091
H	-1.868977	-0.848191	-0.403089
H	-1.868886	0.848190	0.403513

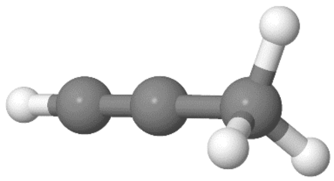

C	-1.436672	0.000000	0.001972
C	-0.185384	0.000001	-0.015980
C	1.239503	0.000001	-0.008542
H	1.632643	-0.911286	-0.468107
H	-2.512352	-0.000001	0.015151
H	1.542385	-0.000045	1.056291
H	1.632646	0.911321	-0.468036

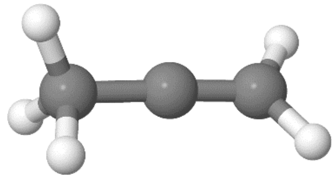

C	0.106120	0.032060	-0.000081
C	-1.303079	0.017351	-0.000001
C	1.380355	-0.005126	0.000006
H	-1.735166	0.415863	0.921360
H	-1.735362	0.415303	-0.921482
H	1.926021	-0.952649	0.000054
H	-1.493099	-1.082758	0.000440
H	1.937232	0.938531	0.000084

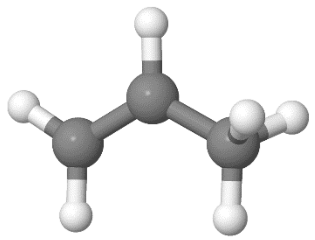

C	-0.073197	0.466554	0.000014
C	-1.320430	-0.212898	-0.000009
C	1.217833	-0.197989	-0.000004
H	-0.096352	1.556345	0.000030
H	1.794781	0.175385	0.863285
H	-2.253173	0.342396	-0.000055
H	-1.354591	-1.298751	0.000072
H	1.169397	-1.284647	0.000058
H	1.794701	0.175266	-0.863394

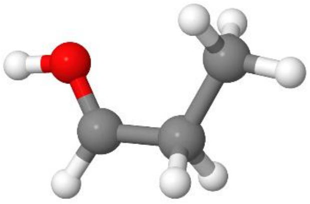

C	0.618587	0.714727	-0.000063
C	-0.822571	0.527203	0.000073
C	1.472578	-0.544896	0.000049
O	-1.315201	-0.638800	-0.000057
H	-1.505551	1.381653	0.000307
H	0.810932	1.378476	-0.861626
H	0.811211	1.378855	0.861108
H	2.524404	-0.260176	-0.000090
H	1.280525	-1.150261	0.887333
H	1.280458	-1.150585	-0.886974
H	-2.291929	-0.649764	0.000040

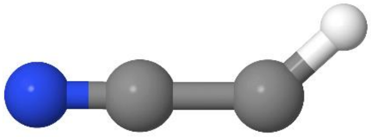

N	1.243120	-0.042937	0.002285
C	-1.236872	0.114418	0.002192
C	0.135108	0.031115	-0.004834
H	-2.091259	-0.572636	-0.000147

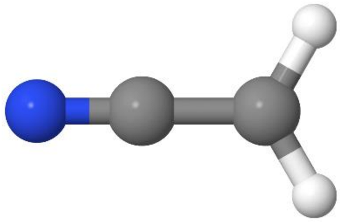

N	-1.366417	-0.000003	-0.000007
C	1.199313	-0.000002	0.000020
C	-0.186731	-0.000003	0.000008
H	1.744771	-0.948059	-0.000059
H	1.744652	0.948111	-0.000059

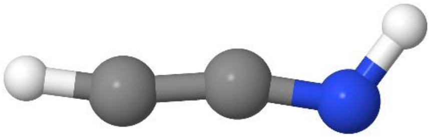

N	1.203139	-0.138889	0.000000
C	-1.290591	-0.026010	0.000000
C	-0.023704	0.054167	0.000000
H	-2.360387	0.116932	0.000000
H	1.824184	0.686346	0.000000

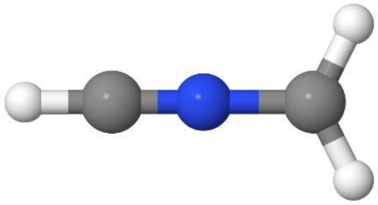

N	-0.132705	0.000001	-0.000009
C	-1.265327	0.000001	-0.000002
C	1.236833	-0.000001	0.000008
H	1.721168	0.965058	-0.000002
H	-2.342436	-0.000005	0.000030
H	1.721166	-0.965061	-0.000002

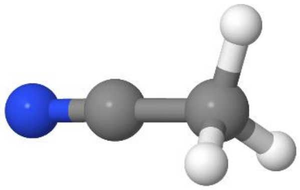

N	-1.444035	-0.000431	0.005264
C	1.167321	0.001056	-0.011777
C	-0.242709	0.001299	-0.017090
H	1.553596	0.937334	-0.430587
H	1.456944	-0.043147	1.063711
H	1.550034	-0.905296	-0.496769

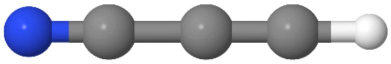

N	-1.874142	0.000687	-0.000019
C	0.617521	-0.000303	0.000134
C	1.832934	0.000194	-0.000171
C	-0.749541	-0.000915	-0.000005
H	2.913508	0.001335	0.000381

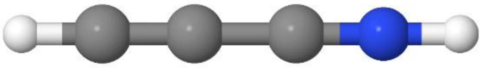

N	1.786308	-0.000858	-0.000085
C	-1.948226	-0.001349	0.000031
C	-0.724141	0.001826	-0.000208
C	0.625351	0.001075	0.000288
H	-3.021485	-0.001393	0.000064
H	2.799421	-0.001909	-0.000130

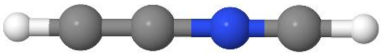

N	0.585601	-0.009465	-0.265691
C	-0.603407	0.009766	0.273737
C	-1.713088	0.027533	0.777186
C	1.643894	-0.026395	-0.745764
H	-2.689086	0.042365	1.220002
H	2.625489	-0.041540	-1.191119

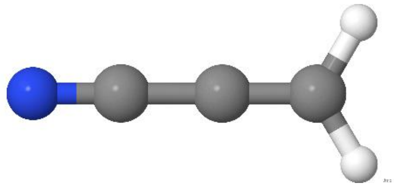

N	-1.996782	-0.000648	0.000300
C	0.541693	0.000202	-0.000180
C	1.815365	-0.000532	0.000253
C	-0.818343	0.000822	-0.000420
H	2.374519	-0.947407	-0.000011
H	2.370666	0.948994	-0.000007

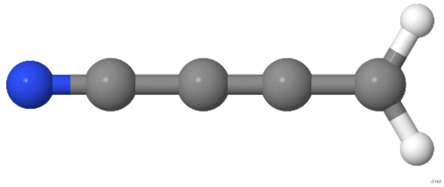

N	-2.643768	-0.000315	0.000861
C	2.493572	-0.000132	0.000545
C	-0.097565	0.000172	-0.000591
C	1.135558	0.000074	-0.000569
C	-1.463111	0.000344	-0.000682
H	3.047602	-0.940956	0.000871
H	3.048052	0.940413	0.000876

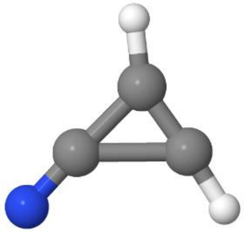

N	-1.623607	-0.106783	-0.000003
C	1.042224	-0.671926	-0.000001
C	0.937624	0.595264	-0.000002
C	-0.494392	0.229788	0.000007
H	1.067332	-1.756335	0.000002
H	1.385177	1.585060	-0.000004

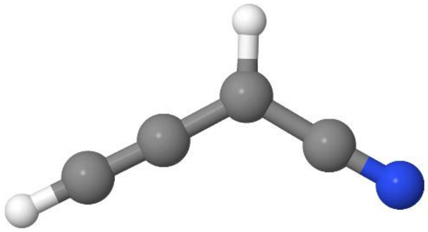

N	2.284512	-0.541179	0.000000
C	-1.166090	0.112248	-0.000006
C	-2.271130	-0.429305	0.000001
C	0.042981	0.754450	0.000002
C	1.253901	0.038236	0.000001
H	-3.232260	-0.914070	0.000011
H	0.082701	1.848545	0.000003

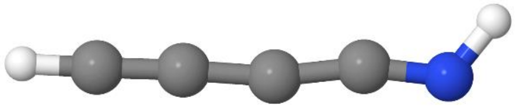

N	2.450994	-0.097693	0.000316
C	-1.358156	-0.004418	-0.000554
C	-0.048469	-0.095590	-0.000290
C	-2.595022	0.036555	0.000393
C	1.236645	0.046698	-0.000087
H	3.101762	0.696057	-0.000154
H	-3.668710	0.088317	0.001163

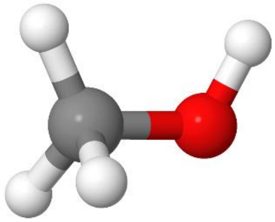

O	0.703691	-0.128567	-0.013648
C	-0.641026	0.048096	-0.025116
H	-0.920744	-0.269584	1.033168
H	-0.978812	1.064637	-0.214304
H	-1.094183	-0.767743	-0.606767
H	1.210365	0.712647	0.047782

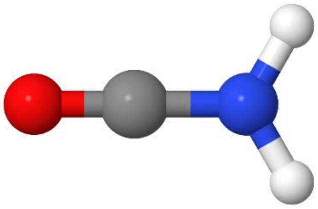

O	-1.286237	-0.000060	-0.000123
N	1.129022	-0.000022	-0.000479
C	-0.148391	0.000148	0.000429
H	1.638599	0.883155	0.000885
H	1.638489	-0.883408	0.000885

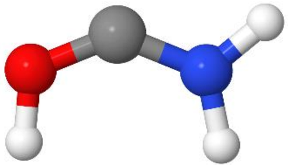

O	-1.213509	0.013377	-0.000060
N	1.141619	0.111292	-0.000054
C	-0.033317	-0.413754	0.000063
H	1.950764	-0.504719	0.000174
H	1.310236	1.117727	-0.000024
H	-1.344363	0.983451	0.000326

References

[1] L. Ziurys, B. Turner, *Astrophys. J. Lett.* **1986**, *302*, L31.

[2] R. C. Woods, T. A. Dixon, R. J. Saykally, P. G. Szanto, *Phys. Rev. Lett.* **1975**, *35*, 1269.

[3] S. Zeng, I. Jiménez-Serra, V. M. Rivilla, J. Martín-Pintado, L. F. Rodríguez-Almeida, B. Tercero, P. de Vicente, F. Rico-Villas, L. Colzi, S. Martín, M. A. Requena-Torres, *Astrophys. J. Lett.* **2021**, *920*, L27.

[4] B. Turner, *Astrophys. J. Lett.* **1977**, *213*, L75.

[5] F. J. Lovas, J. Hollis, A. J. Remijan, P. Jewell, *Astrophys. J.* **2006**, *645*, L137.

[6] A. Belloche, R. Garrod, H. Müller, K. Menten, C. Comito, P. Schilke, *Astron. Astrophys.* **2009**, *499*, 215.

[7] W. M. Irvine, P. Friberg, A. Hjalmarson, S. Ishikawa, N. Kaifu, K. Kawaguchi, S. C. Madden, H. E. Matthews, M. Ohishi, S. Saito, H. Suzuki, P. Thaddeus, B. E. Turner, S. Yamamoto, L. M. Ziurys, *Astrophys. J.* **1988**, *334*, L107.

[8] D. Buhl, L. E. Snyder, The Detection of MM-Wave Transition of Methlacetylene, in M. A. Gordon, L. E. Snyder (Editors), *Mol. Galact. Environ.*, page 187, John Wiley and Sons, New York, NY **1973**.

[9] P. Solomon, K. Jefferts, A. Penzias, R. Wilson, *Astrophys. J.* **1971**, *168*, L107.

[10] B. Turner, *Astrophys. J.* **1971**, *163*, L35.

[11] M. Ohishi, S.-i. Ishikawa, T. Amano, H. Oka, W. M. Irvine, J. E. Dickens, L. Ziurys, A. Apponi, *Astrophys. J.* **1996**, *471*, L61.

[12] H. Gupta, C. Gottlieb, V. Lattanzi, J. Pearson, M. McCarthy, *Astrophys. J. Lett.* **2013**, *778*, L1.

[13] J. A. Ball, C. A. Gottlieb, A. Lilley, H. Radford, *Astrophys. J.* **1970**, *162*, L203.

[14] E. Dzib, J. L. Cabellos, F. Ortíz-Chi, S. Pan, A. Galano, G. Merino, *International Journal of Quantum Chemistry* **2019**, *119*, e25686.
